# Sulphated alumina tungstic acid (SATA): a highly efficient and novel heterogeneous mesostructured catalyst for the synthesis of pyrazole carbonitrile derivatives and evaluation of green metrics†

**DOI:** 10.1039/c9ra09013d

**Published:** 2020-01-03

**Authors:** Ryhan Abdullah Rather, Mohd Umar Khan, Zeba N. Siddiqui

**Affiliations:** Department of Chemistry, Aligarh Muslim University Aligarh 202002 India siddiqui_zeba@yahoo.co.in zns.siddiqui@gmail.com

## Abstract

A novel mesostructured catalyst sulphated alumina tungstic acid (SATA) has been prepared by an easy route. Various techniques such as IR, XRD, SEM, EDX, TEM, TGA and BET were used to characterize the synthesized catalyst. The catalytic activity of the meso material has been explored by synthesizing a series of new pyrazole carbonitrile derivatives from aromatic aldehydes, ethylcyanoacetate, phenylhydrazine/hydrazine hydrate in ethanol under reflux conditions. Furthermore, the “greenness” of this protocol when estimated by green metrics, displayed satisfactory results. The protocol is free from column chromatography, and toxic solvents and is more efficient as compared to reported ones.

## Abstract 2

A novel mesostructured catalyst sulphated alumina tungstic acid (SATA) has been prepared by an easy route.

## Graphical abstract

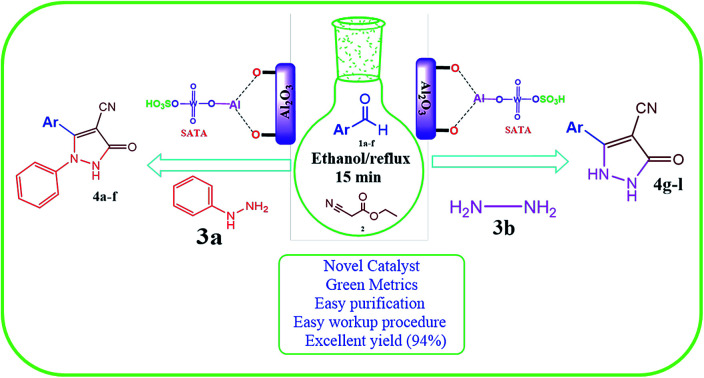

## Introduction

Various eco-friendly sulfonic acid-based materials have been successfully used as catalysts in organic synthesis^1^ due to their higher acidic strength, increased thermal stability, reduced toxicity, *etc.*^2^ Various sulfonic acids have been heterogenized on solid supports to meet sustainability requirements such as recyclability, increased efficiency in terms of higher surface area/large pore space *e.g.*, sulfonated silica materials,^3^ sulfonated carbon materials,^4^ sulphated zirconia,^5^ sulphated hybrid materials,^6^ sulfonated magnetic materials,^7^*etc.*^8^ Solid acids are one of the most important classes of catalyst used in the chemical industry.^2,3,9^ Compared to liquid acids, solid acids cause less corrosion of the facility,^10–12^ can be easily separated from the reaction medium for recycling,^13–15^ and their properties may be tunable for specific feedstock and products.^16–18^ Among the solid acids, a great deal of attention has been paid to tungstic acid, as it had shown considerable superiorities, especially in the heterogeneous form in terms of acidity, thermal stability, and hydrophobicity, in comparison with the other materials,^19,20^ and thus hold a promising candidate for application in various organic reactions. Basic reactions such as hydrodesulfurization, hydrodenitrogenation, and hydrodearomatisation are the most important reactions catalyzed by tungstic acid in the chemical industry.^21,22^ Since then, tungstic acid has received continuous attention because of its increasing advantages in catalysis. Other organic reactions catalysed by tungstic acid include oxidation,^23^ hydroxylation,^24^ epoxidation^25^ and many other transformations.^26–28^ In spite of these advantageous properties and applications, tungstic acid suffer from severe drawbacks such as their deactivation during reaction due to coke formation on the surface and low surface area.^29^ This limits its applications in many reactions.^30^ These drawbacks, however, can be removed by dispersing them on solid support materials with a high surface area. In this regard, mesoporous materials are good candidates as they possess unique structural features such as huge BET, large surface area, tunable pore diameter, flexibility to accommodate several functional groups and metals on to the surface. The introduction of mesoporous materials has brought tremendous economic and environmental revolution^31^ and plays a key role in catalysis^32^ at the industrial scale. Among mesoporous materials, mesoporous alumina is a good choice as it is cheap and easily available and also possesses beneficial properties such as high thermal stability, high purity, favourable bulk density, *etc.*^33^

In the present work, we have developed an easy route for the synthesis of mesoporous sulphated alumina tungstic acid and explored its catalytic activity by synthesizing a series of new pyrazole carbonitrile derivatives *via* multicomponent reaction. It is worthy to mention that pyrazole carbonitriles are interesting scaffolds due to their potential biological activities including anti-HIV^34^ antibacterial and antifungal^35^ anticancer^36^ anti-depressant activities^37^*etc.*

## Results and discussion

**Scheme 1:**
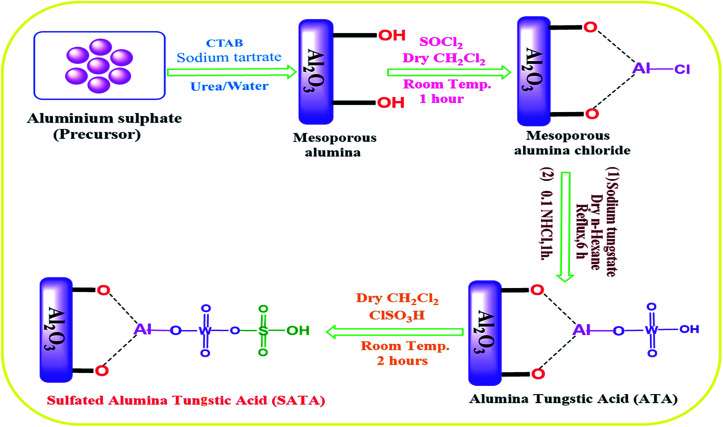
Flow diagram depicting preparation of catalyst.

The synthesis of catalyst sulphated alumina tungstic acid (SATA) is shown in Scheme 1. H^+^ ion concentration of the catalyst was obtained as 0.44 meq. g^−1^ using back titration method.

**Fig. 1:**
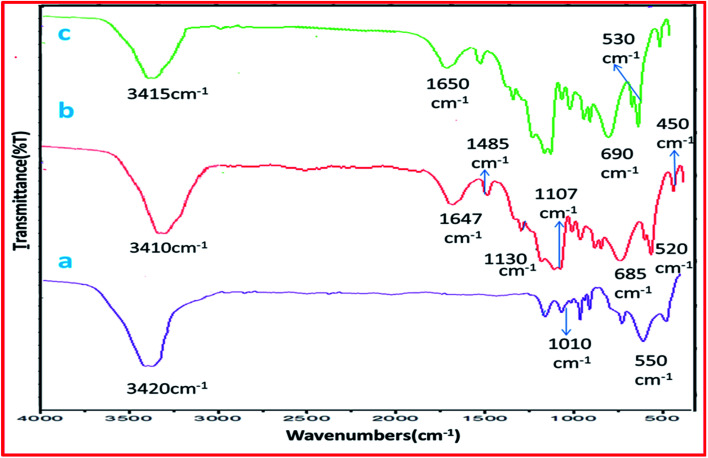
FT-IR spectra of (a) mesoporous alumina (b) fresh SATA and (c) recycled SATA.

The IR spectrum (Fig. 1a), exhibited absorption peak at 3420 cm^−1^ due to surface OH groups. The absorption peaks between 510–1000 cm^−1^ were due to bending and stretching vibrations of Al–O bond.^38^ Sulphated alumina tungstic acid (SATA) (Fig. 1b) showed bands at 1647 cm^−1^ due to WO_4_^2−^ group, 1130 cm^−1^ due to SO_2_^2−^ group and 600–700 cm^−1^ due to S–O bond.

**Fig. 2:**
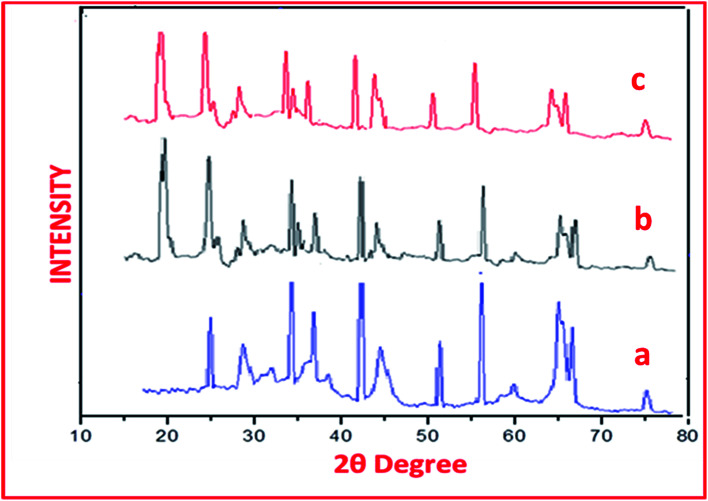
XRD spectra of (a) mesoporous alumina, (b) fresh SATA and (c) recycled SATA.

In the XRD spectrum of catalyst sulphated alumina tungstic acid (SATA) (Fig. 2a) peaks at 29.89°, 44.96°, 59.95°, 64.99°, 69.92° corresponded to γ-Al_2_O_3_ and peaks at 24.98°, 34.95°, 39.98°, 50.45°, 54.89° were due to different phases of α-Al_2_O_3_.^39^ The XRD spectrum of SATA exhibited an additional peak at 22° (Fig. 2b) due to WO_4_ group.^40^ Some insignificant changes in the nature of peaks were observed because of bonded sulfonic acid groups on the alumina surface.

**Fig. 3:**
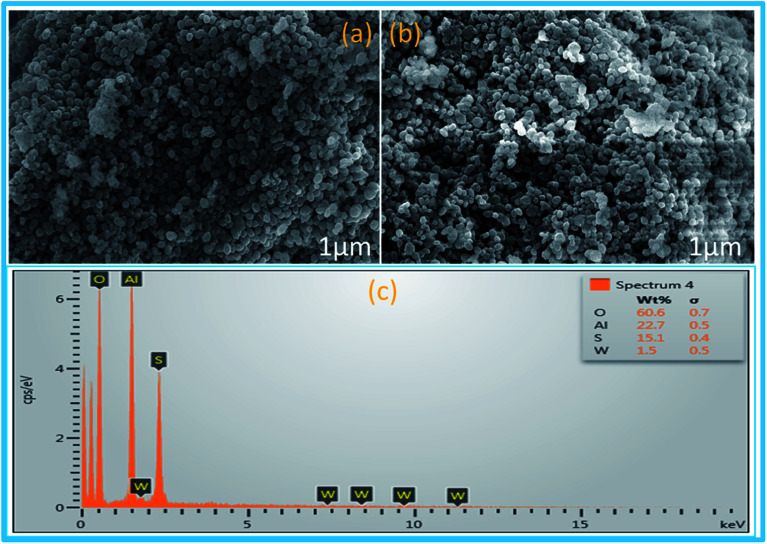
SEM images of (a) freshly synthesized mesoporous Al_2_O_3_, (b) SATA and (c) EDX analysis of SATA.

**Fig. 4:**
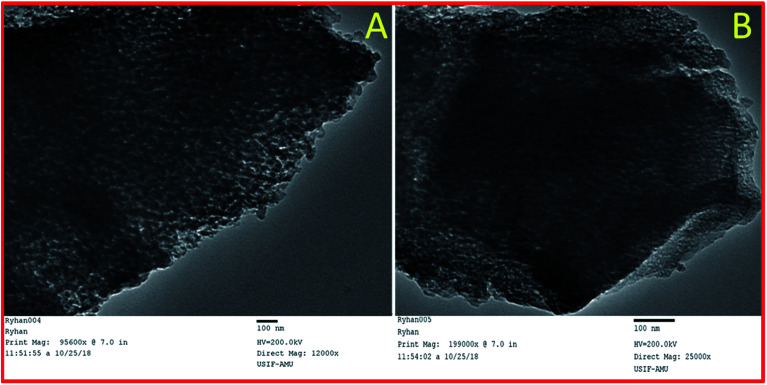
TEM images of (A) fresh catalyst SATA and (B) recycled catalyst SATA.

SEM images of synthesized mesoporous alumina (Fig. 3a) showed the spherical shape of the synthesized mesoporous Al_2_O_3_ particles. After functionalization, there were insignificant morphological changes in the synthesized material sulphated alumina tungstic acid (Fig. 3b). The EDX analysis (Fig. 3c) of the catalyst showed the presence of constituent elements W, Al, O, and S. TEM image (Fig. 4A) of the catalyst (SATA) exhibited a wormhole-like porous structure. Furthermore the TEM image of the recycled catalyst (Fig. 4B) showed same morphology indicating that the structure of the synthesized catalyst was not disturbed.

**Fig. 5:**
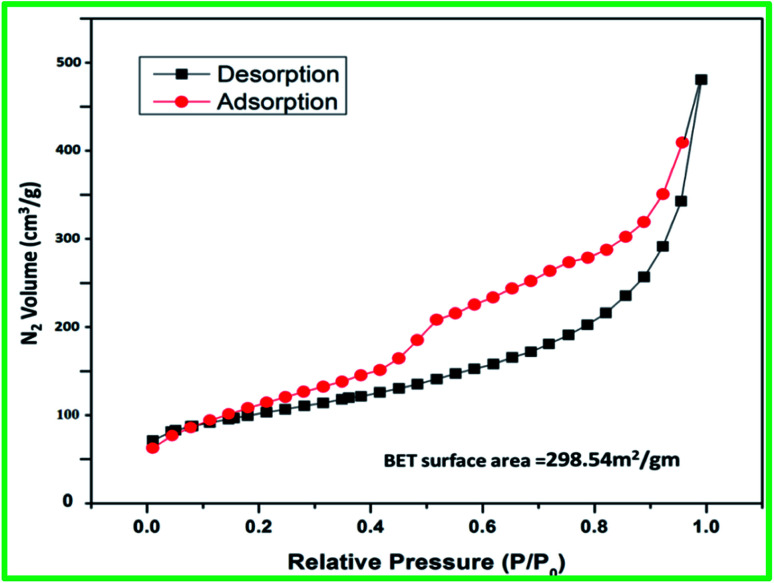
BET analysis of synthesized catalyst SATA.

The surface area of catalyst (SATA) was found to be 298.54 m^2^ g^−1^ (Fig. 5) which was slightly lower than the mesoporous alumina (308 m^2^ g^−1^).^41^ The decrease in the surface area could be due to functionalization of mesoporous alumina.

**Fig. 6:**
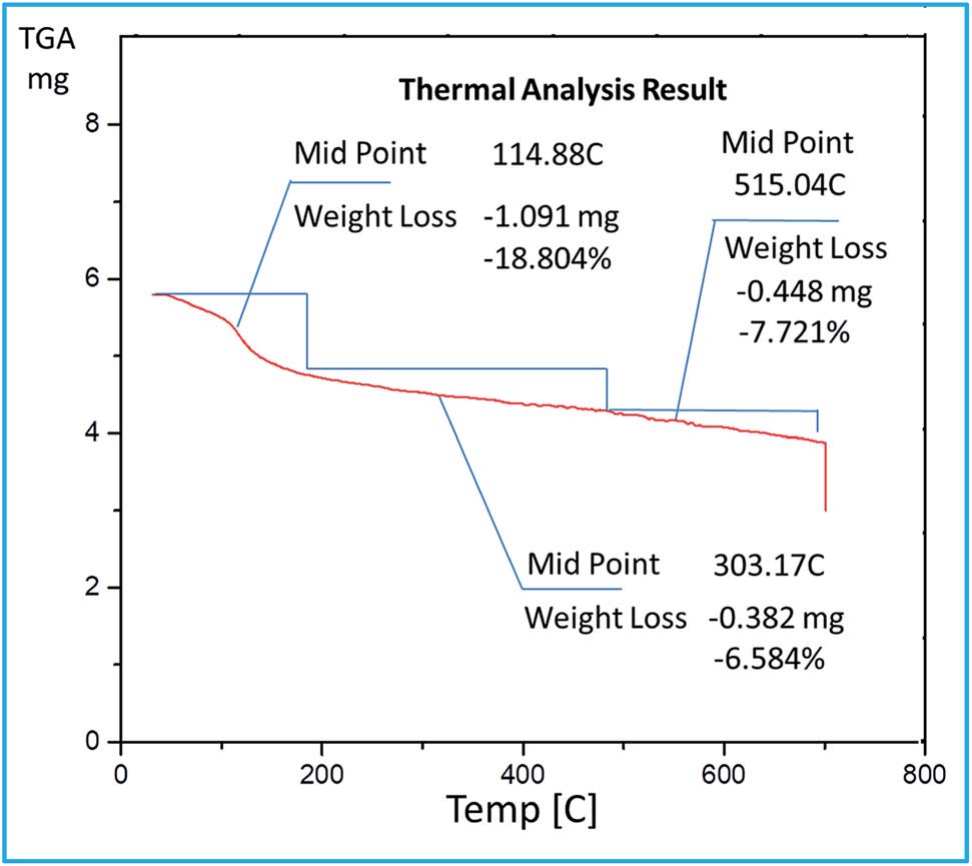
TGA curve of synthesized catalyst SATA.

The TGA curve (Fig. 6) showed a weight loss of 18.8% up to 114 °C due to removal of water molecules trapped in alumina framework. Another weight loss of 6.58% at 303 °C can be attributed to the decomposition of bonded sulfonic acid groups from the surface of mesoporous alumina.

### Optimization of reaction conditions

To find out suitable reaction conditions using SATA for the synthesis of pyrazole carbonitriles, various parameters such as different catalysts/solvents, catalyst loading, temperature *etc.* were tried to get maximum yield of product (4a). To find suitable catalyst for our reaction we first tried the reaction in the absence of any catalyst. The reaction failed to produce any yield indicating the need of a catalyst (Table 1, entry 1). Then effect of the different sulphuric acid based catalysts under reflux condition was examined. Among them, CH_3_COOH and H_2_SO_4_ catalyzed reactions did not give satisfactory result (Table 1, entries 2 and 3). Among silica, alumina and zirconia-based sulphuric acids, H_2_SO_4_–alumina afforded slightly better product yield (Table 1, entry 6). Similarly, PEG and xanthan like supported sulphuric acid organic polymers also gave unsatisfactory yields (Table 1, entries 8 and 9). In the presence of sodium tungstate, the product yield was slightly increased but took a longer time (Table 1, entry 10). The use of alumina tungstic acid (ATA) showed better results (Table 1, entry 11). However, the best results were obtained with sulphated ATA (SATA) affording 94% product yield in 15 min (Table 1, entry 12). Then our study focused on the development of the optimal reaction conditions for this transformation, which included solvent screening and influence of the catalyst amount. Several organic solvents like isopropanol, acetonitrile, methanol, hexane, toluene, and ethane diol when tested, (Table 1, entries 19–24), ethanol was found as a preferred solvent for maximum conversion of substrate into products (94%) in the minimum time period (15 min) (Table 1, entry 12). To find the optimized amount of the catalyst, the reaction was carried out by varying the amount of the catalyst on the model reaction (Table 2). It was found that the conversion of the pyrazole carbonitrile derivative increased linearly with increase in the amount of catalyst from 50–100 mg (Table 2, entry 1–3). With further increase in the amount of catalyst, the product yield was not increased, so 100 mg of the catalyst was found to be the optimum amount of catalyst for the desired reaction. The temperature effect played a vital role in the synthesis of pyrazole carbonitriles. The reaction was unsuccessful at room temperature while increasing it upto 80 °C product formation also increased linearly and completed at 80 °C (Table 3). As indicated in Table 3, the effect of increasing temperature (from 25 to 80 °C) is directly related to the improved yield of the product. Upon increasing the temperature further, the reaction afforded lesser yield of product. Thus, 80 °C was found to be the optimum temperature for the reaction.

**Table 1:** Effect of various reaction media for the model reaction^a^

Entry	Catalyst	Condition	Time^b^	Yield^c^	References^d^
1	—	Ethanol/reflux	18 h	—	
2	AcOH (10 mol%)	Ethanol/reflux	9 h	56	42
3	H_2_SO_4_ (10 mol%)	Ethanol/reflux	8 h	54	43
4	H_2_SO_4_–SiO_2_ (200 mg)	Ethanol/reflux	5 h	62	44
5	H_2_SO_4_–ZrO_2_ (200 mg)	Ethanol/reflux	6 h	58	45
6	H_2_SO_4_–Al_2_O_3_ (200 mg)	Ethanol/reflux	3.5 h	70	46
7	H_2_SO_4_–cellulose (200 mg)	Ethanol/reflux	6.5 h	60	47
8	H_2_SO_4_–zanthan (200 mg)	Ethanol/reflux	6 h	56	48
9	H_2_SO_4_–PEG (200 mg)	Ethanol/reflux	5.2 h	64	49
10	Na_2_WO_3_ (10 mol%)	Ethanol/reflux	4 h	68	50
11	ATA (200 mg)	Ethanol/reflux	92 min	76	Present work
**12**	**ATA–OSO**_**3**_ **H(SATA) (100 mg)**	**Ethanol/reflux**	**15 min**	**94**	**Present work**
13	FeCl_3_ (10 mol%)	Ethanol/reflux	6.2 h	42	51
14	ZnCl_2_ (10 mol%)	Ethanol/reflux	6 h	40	52
15	ZnO (10 mol%)	Ethanol/reflux	7 h	24	53
16	MgO (10 mol%)	Ethanol/reflux	7 h	26	54
17	SATA (100 mg)	Solvent free	10 h	Traces	
18	SATA (100 mg)	Water	7 h	52	
19	SATA (100 mg)	Methanol	7 h	78	
20	SATA (100 mg)	Isopropanol	7.2 h	72	
21	SATA (100 mg)	Acetonitrile	7 h	56	
22	SATA (100 mg)	Hexane	8 h	52	
23	SATA (100 mg)	Toluene	7.2 h	56	
24	SATA (100 mg)	Ethylene glycol	7 h	48	

a Reaction conditions: 4-chlorobenzaldehyde 1a (1 mmol), ethyl cyanoacetate 2 (1 mmol), phenylhydrazine 3a (1 mmol), in presence of different reaction media.

b Reaction progress monitored by TLC.

c Isolated yield.

d References of the catalysts used before for the similar kind of reactions under different reaction conditions.

**Table 2:** Effect of catalyst loading on the model reaction^a^

Entry^a^	Catalyst loading (mg)	Time^b^ (min)	Yield^c^ (%)
1	30	52	70
2	50	45	78
3	100	15	94
4	150	15	88
5	200	15	88

a Reaction conditions: 4-chlorobenzaldehyde 1a (1 mmol), ethyl cyanoacetate 2 (1 mmol), phenylhydrazine 3a (1 mmol), in presence of different amounts of SATA in ethanol under reflux condition.

b Reaction progress monitored by TLC.

c Isolated yield.

**Table 3:** Effect of temperature on model reaction^a^

Entry	Temperature (°C)	Time^b^	Yield^c^ (%)
1	25	24 h	No reaction
2	45	1.5 h	40
3	65	60 min	75
4	75	45 min	82
5	80	15 min	94
6	100	24 min	90

a Reaction conditions: 4-chlorobenzaldehyde 1a (1 mmol), ethyl cyanoacetate 2 (1 mmol), phenylhydrazine 3a (1 mmol), in presence of SATA (100 mg) in 5 mL ethanol/reflux.

b Reaction progress monitored by TLC.

c Isolated yield.

In the light of green chemistry, it was vital to certify our procedure as eco-friendly, which was accomplished in terms of atom economy (AE), reaction mass efficiency (RME), overall efficiency (OE), carbon efficiency (CE), process mass intensity (PMI), *E*-factor and solvent intensity (SI).^55–58^ Details of reactants and products used for metric calculations are given in ESI.† The calculated values for AE (76–83%), OE (91–94%), CE (83–89%), *E*-factor (19–12), SI (19–12) and PMI (20–13) are summarized in (Table 4) which endorse this presented method as green and sustainable.

**Table 4:** Summary of green metrics calculations of the synthesized compounds (4a–l)

Entry	% yield	% AE^a^	% CE^b^	% RME^c^	% OE^d^	PMI^e^	SI^f^	*E*-factor^g^
4a	94	81.73	88.88	76.84	94.01	15.47	14.17	14.47
4b	94	82.25	88.88	77.41	94.11	14.97	13.68	13.97
4c	94	82.25	88.88	77.41	94.11	14.97	13.68	13.97
4d	92	81.73	88.88	75.17	91.97	15.81	14.48	14.81
4e	94	83.73	88.88	78.76	94.06	13.58	12.31	12.58
4f	92	81.50	89.47	74.90	91.90	16.03	14.70	15.03
4g	94	78.86	83.33	72.25	94.00	20.46	19.08	19.46
4h	92	77.69	83.33	71.47	91.99	19.98	18.58	18.98
4i	94	77.69	83.33	73.02	93.98	19.57	18.21	18.57
4j	92	76.86	83.75	70.71	91.99	20.91	19.50	19.91
4k	92	89.13	83.33	82.01	92.01	17.43	16.21	16.43
4l	94	76.50	84.61	71.91	94.00	20.86	19.47	19.86

a % AE: percentage atom economy.

b % CE: percentage carbon efficiency.

c % RME: percentage reaction mass efficiency.

d % OE: percentage overall efficiency.

e PMI: process mass intensity.

f SI: solvent intensity.

g *E*-factor. Catalyst amount was not used in the calculations because of its reusability.^47^ Calculations up to the crude product in all cases.

### Catalytic reaction

**Scheme 2:**
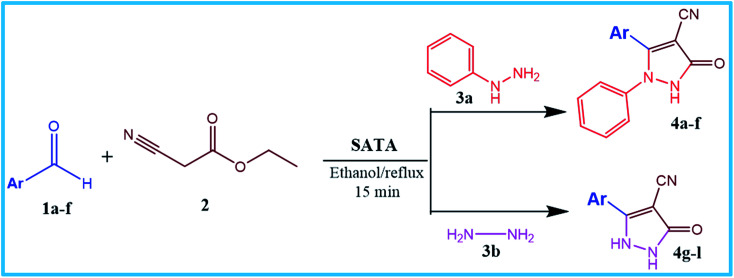
General scheme for the formation of pyrazole carbonitrile derivatives.

After optimizing the reaction conditions, a variety of pyrazole carbonitriles were synthesized (Table 5) (Scheme 2). The results indicated that reaction proceeded smoothly in the presence of a catalyst (SATA) giving an excellent yield of products successfully within a shorter period of time.

**Table 5:** Synthesis of pyrazole carbonitrile derivatives in presence of SATA under reflux conditions^a^

Entry	1a–f	Products	Time^b^ (min)	Yield^c^ (%)
1	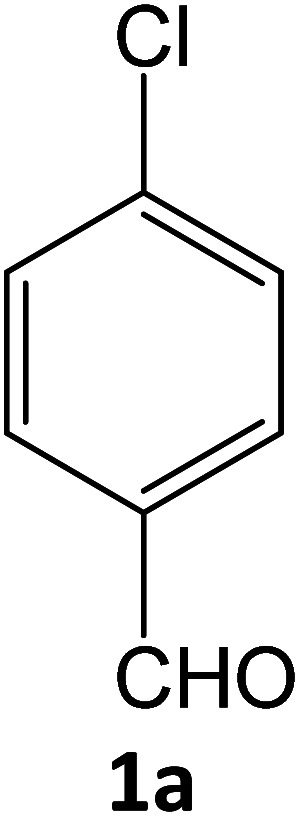	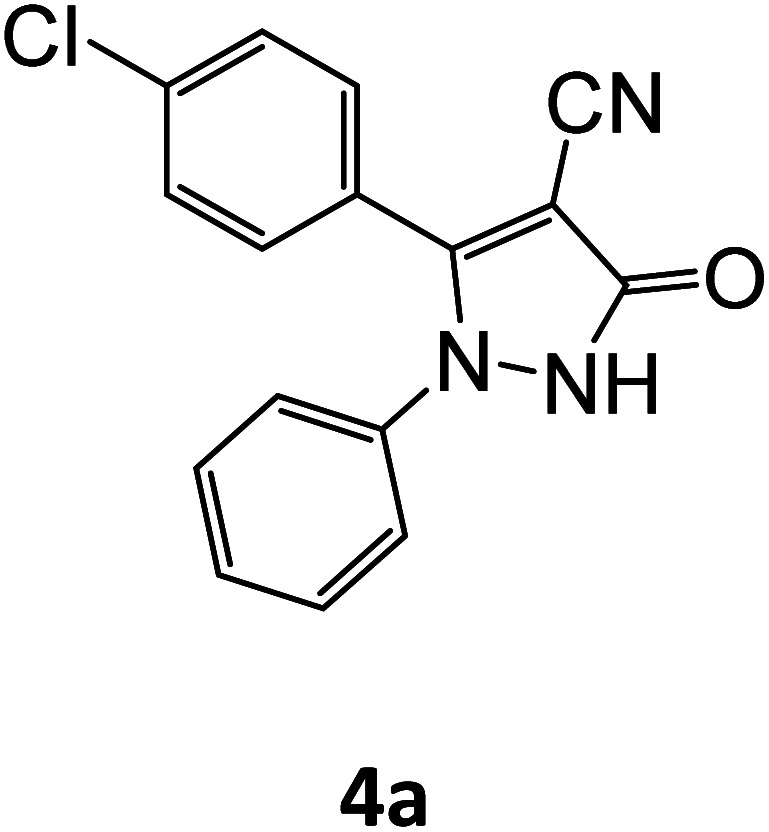	15	94
2	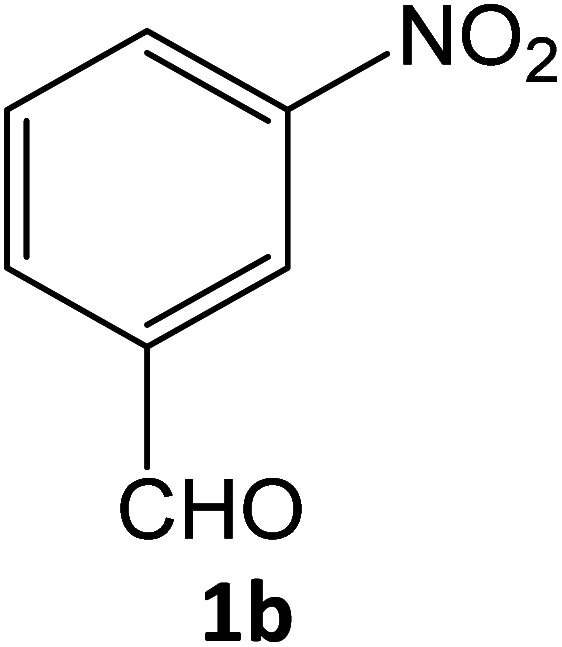	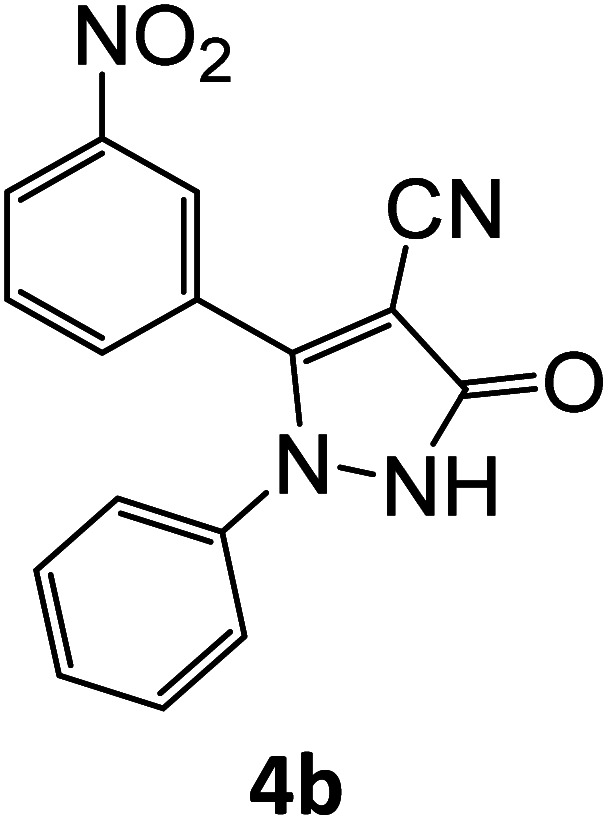	16	94
3	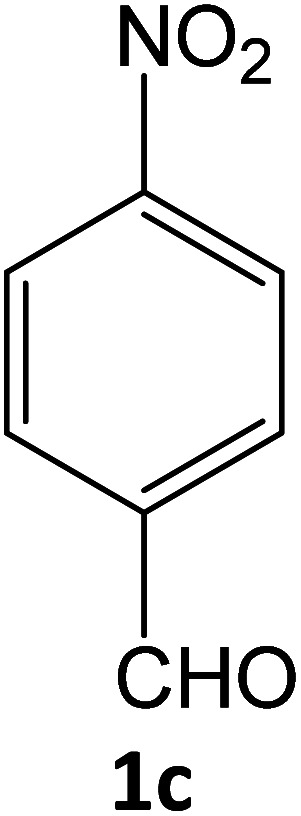	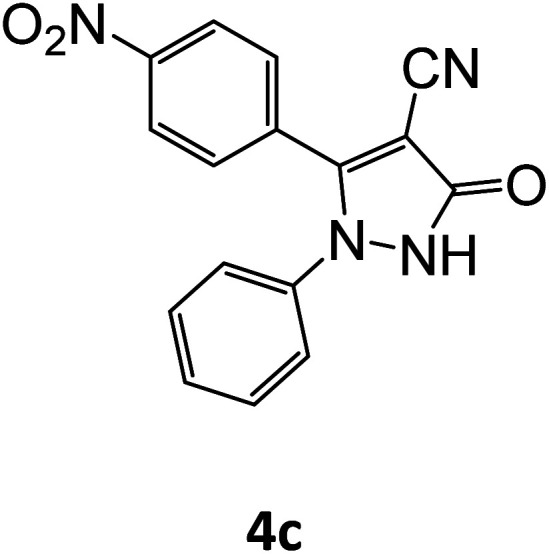	15	94
4	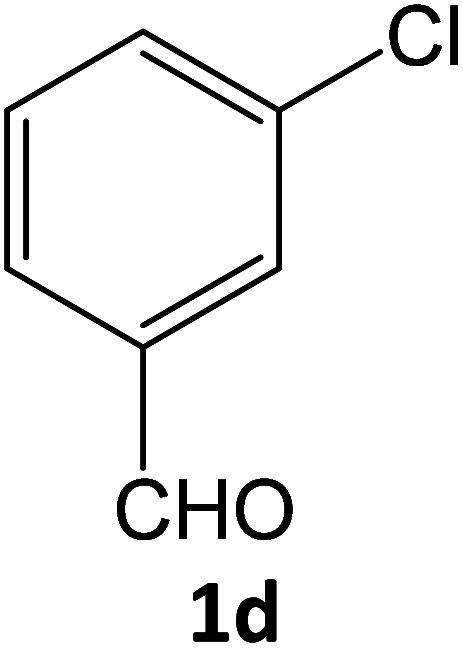	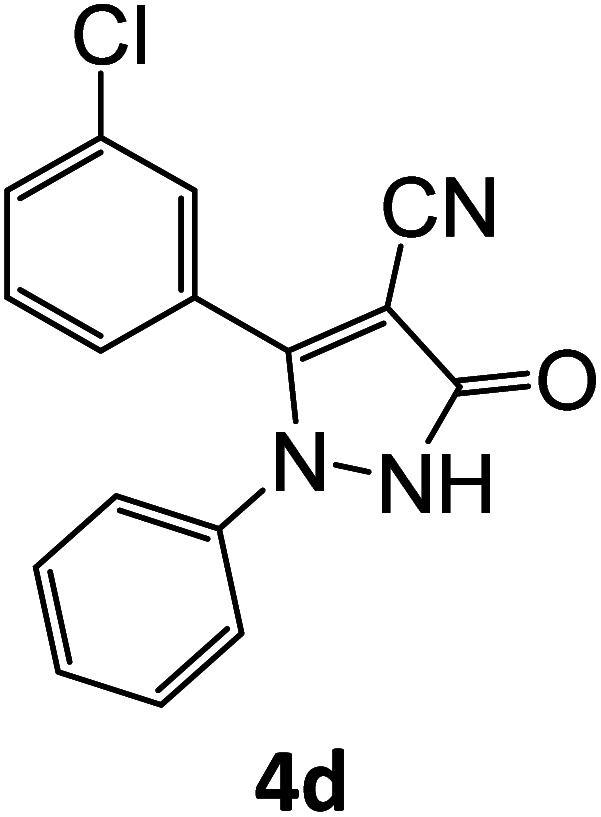	16	92
5	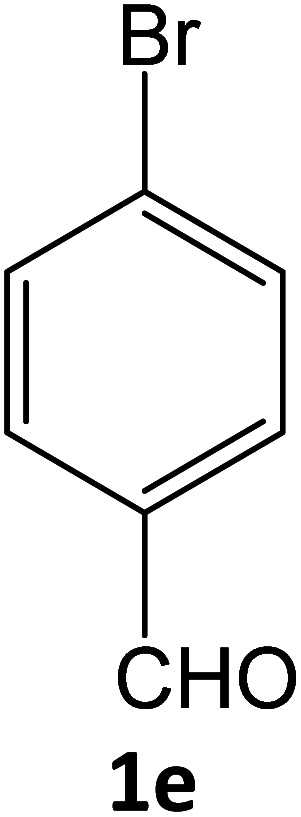	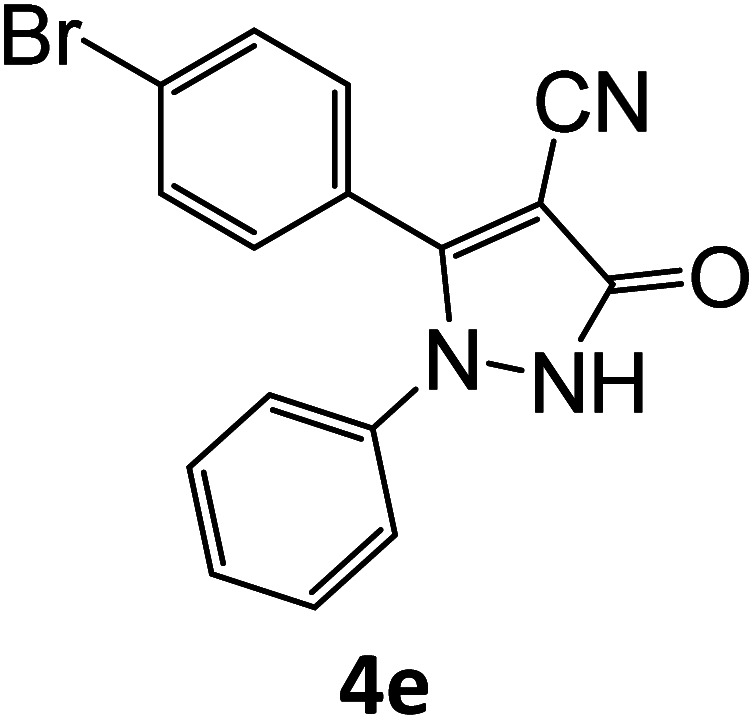	15	94
6	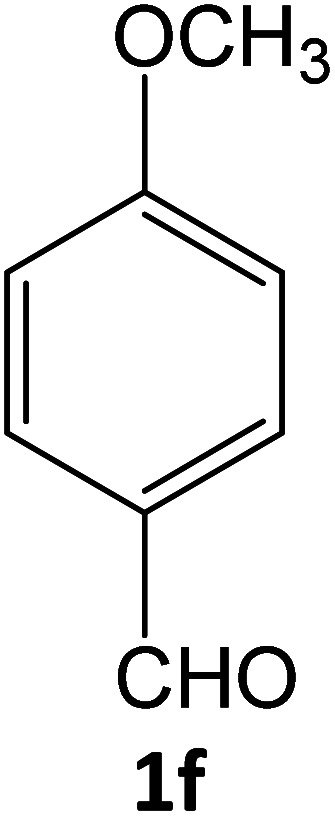	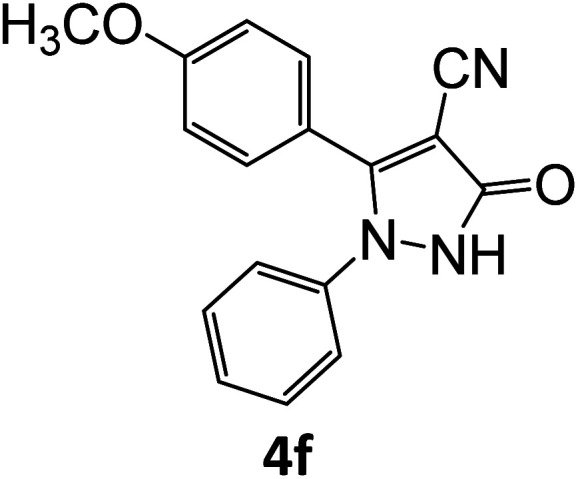	15	92
7	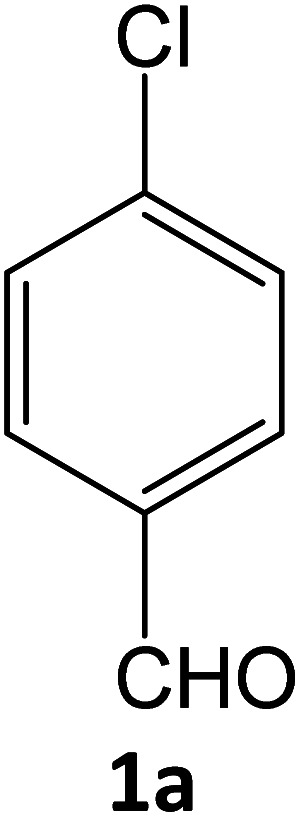	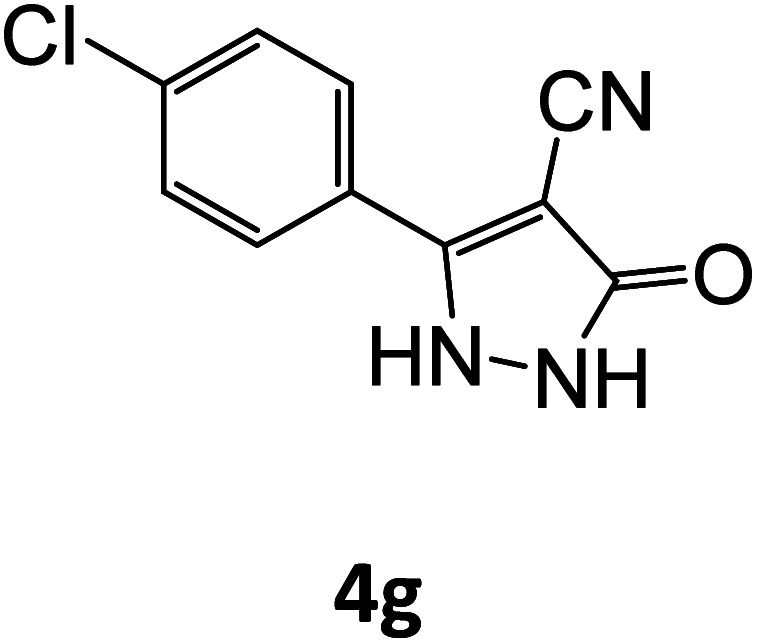	15	94
8	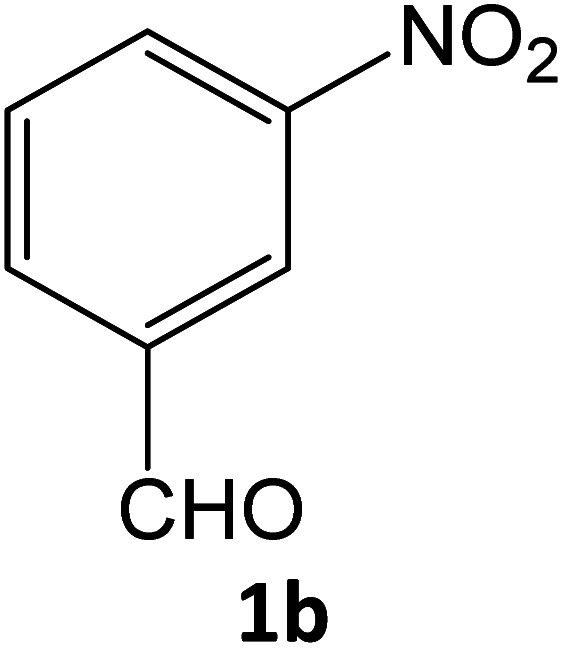	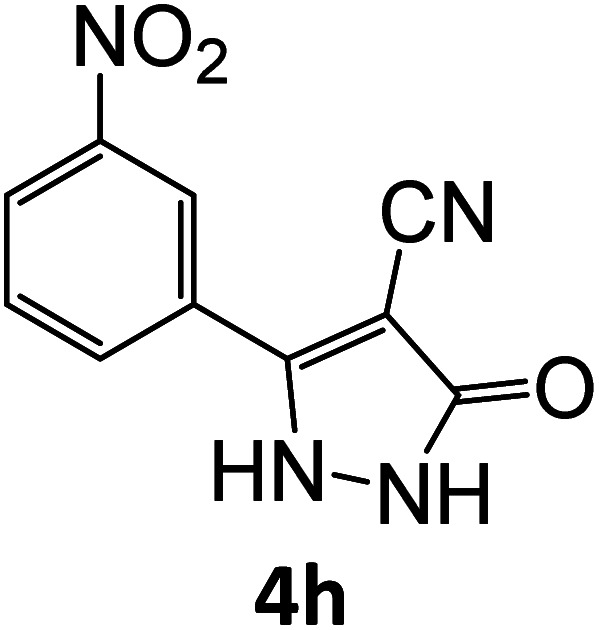	17	92
9	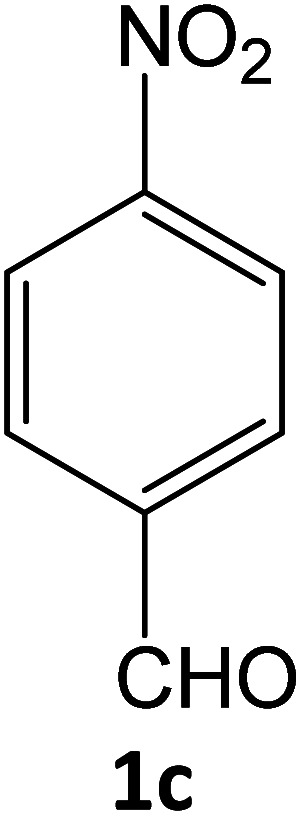	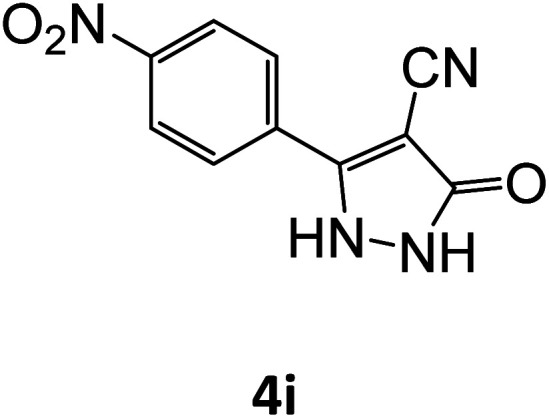	15	94
10	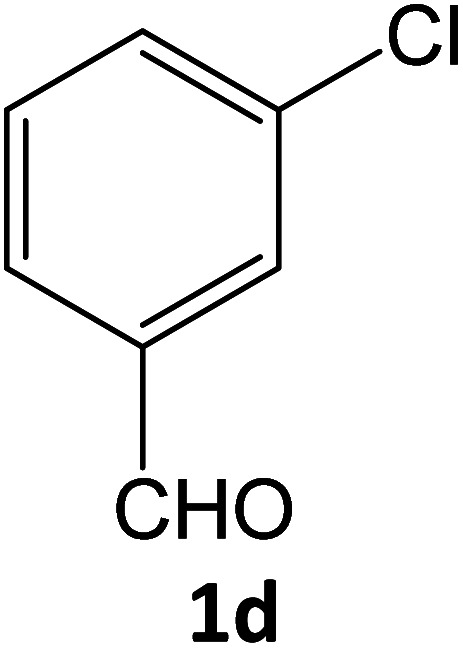	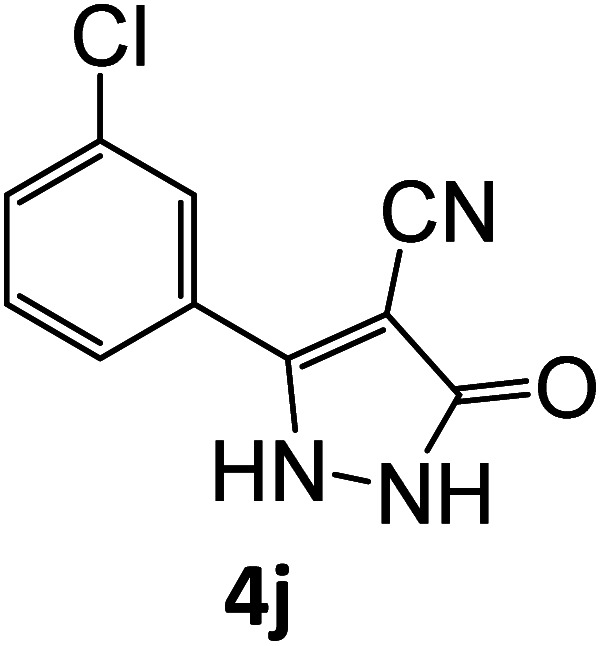	16	92
11	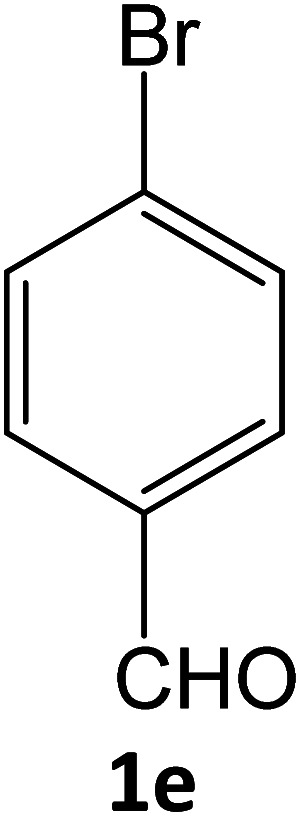	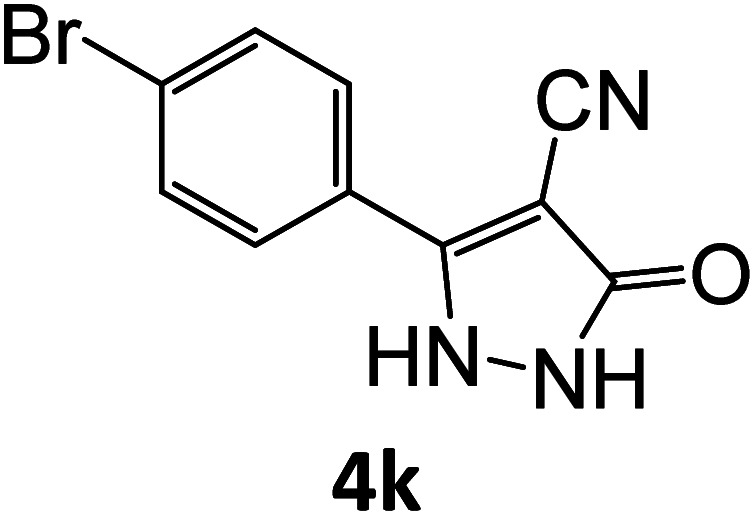	15	92
12	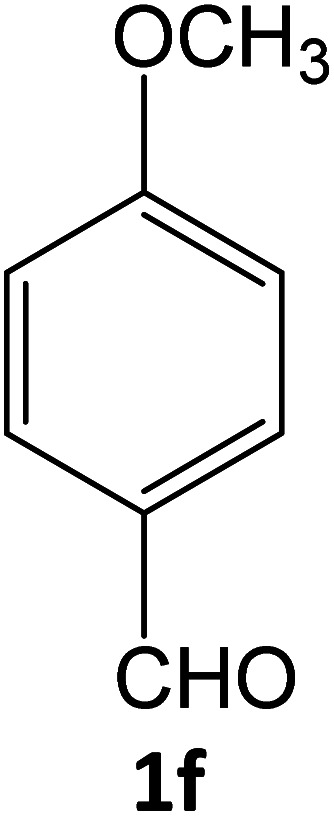	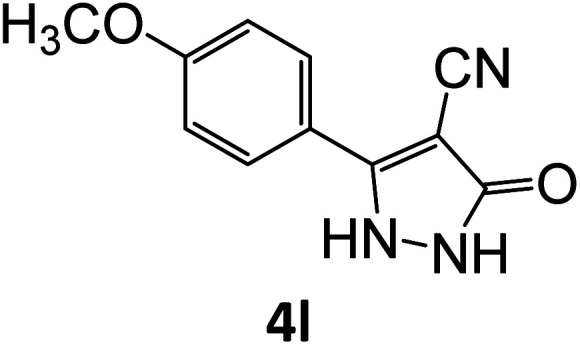	15	94

a Reaction of aldehydes (1a–f) (1 mmol), ethylcyanoacetate (2) (1 mmol) and hydrazines (3a/3b) in presence of SATA (100 mg) in 5 mL ethanol/reflux.

b Reaction progress was monitored by TLC.

c Isolated yield.

In order to demonstrate the advantage of our protocol, a comparison with reported available protocols in the literature was done (Table 6). In comparison to reported procedures, the present protocol proved competent, environmentally benign and practically more sound (Table 6). Moreover, catalyst can be easily separated at the end of reaction and has shown good reusability.

**Table 6:** Comparison of efficiency of sulphated alumina tungstic acid (SATA) with reported methods

Entry	Catalyst/material	Reaction condition	Reaction medium	Yield (%)	Time	Reference
1	SATA	Reflux	Ethanol	94	15 min	Present work
2	CuO/ZrO_2_	Stirring, 40 °C	Water	88	1.5 h	59
3	Fe_3_O_4_@Si@MoO_2_	RT	Solvent free	88	30 min	60
4	Ti(NMe_2_)_2_(pypyr)_2_	Reflux, 100 °C	Toluene	71	36 h	61
5	(Bim)OH	Grinding	Water	75	30 min	62
6	Yb(PFO)_3_	120 °C	Solvent free	76	1.5 h	63

### Reusability and heterogenity of the catalyst

**Fig. 7:**
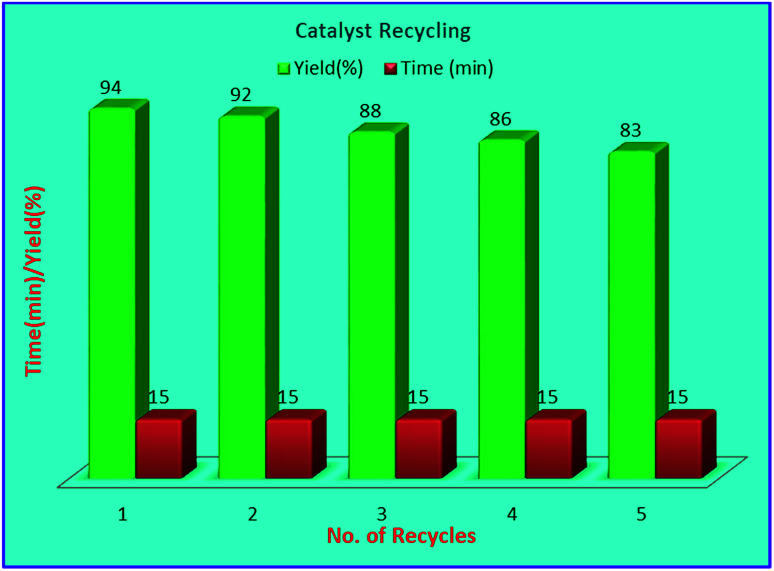
Recycling data of the catalyst.

The recycling experiments were performed to explore the recyclability level of our catalytic scheme. The recyclability level was studied for the heterogeneous nature of the catalyst (Fig. 7). The recovered catalyst catalysed the formation of 4a successfully for five consecutive cycles with good activity. The morphology of the recovered catalyst was almost unchanged as shown by XRD and TEM analysis (Fig. 2c and 4b) respectively. Furthermore, to figure out the heterogeneity of the catalyst sulphated alumina tungstic acid (SATA), hot filtration test was accomplished. The model reaction containing 4-chlorobenzaldehyde (1a), ethyl cyanoacetate (2), phenylhydrazine (3a) and (SATA) (100 mg) in ethanol was refluxed for 13 min and filtered off while hot. Taking this filtrate as reaction mixture, the reaction was further continued under similar reaction conditions for 30 min and it was found that the reaction could not proceed further. Therefore, it was concluded that the catalyst was heterogeneous in nature.

## Conclusion

Herein, a good catalytic system based on mesoporous alumina was synthesized. The heterogeneous catalyst displayed excellent activity for the synthesis of pyrazole carbonitriles. All the substrates could be easily converted to products with high yield. The distinguishing features of this method included easy separation of the catalyst, mild reaction conditions, enhanced reaction rates, cleaner reaction profiles, and easy product separation procedures, which make this method attractive. Furthermore, the method described produced negligible waste, with good to excellent values of green chemistry metrics (AE, CE, RME, OE, PMI, SI, and *E*-factor), and showed standard choice in this framework.

## Experimental aspect

### Preparation of mesoporous alumina (Al_2_O_3_) and alumina tungstic acid (ATA)

Mesoporous alumina was synthesized according to reported procedure^64^ with slight modifications. Cetrimonium bromide (CTAB) (0.2 mmol), aluminium sulphate (Al_2_(SO4)_3_·18H_2_O) (1 mmol), urea (CO(NH_2_)_2_) (4 mmol), and sodium tartrate (C_4_H_4_O_6_Na_2_) (0.7 mmol) were suspended in demineralized water for about 0.5 h until complete dissolution. The solution was then kept in an autoclave with Teflon lining with maintained temperature at 165 °C for 8 h. The white precipitate as formed, after allowing autoclave to cool was collected, washed carefully with distilled water, dried at 80 °C for 12 h and finally calcined at 550 °C for 3 h to get rid of the impurities.

### Preparation of alumina tungstic acid (ATA)

Dropwise addition of thionyl chloride (SOCl_2_ 20 g) to synthesized mesoporous alumina (20 g) in dry CH_2_Cl_2_ (50 mL) at ambient temperature with constant stirring was performed. HCl and SO_2_ gases were evolved and reaction was allowed to proceed for another 1 h. After that CH_2_Cl_2_ (solvent) was removed from the reaction mixture under suction to obtain alumina chloride. In next step mesoporous AlCl_3_ (6.00 g) as formed and Na_2_WO_4_ (7.03 g) were taken in solvent *n*-hexane (10 mL) and refluxed with stirring for 6 h. The solid as formed was filtered out from reaction mixture, washed with water, dried and stirred for another 1 h with 0.1 N HCl (40 mL) (Scheme 1). Finally, the solid was removed from the reaction mixture by filtration, washed several times with distilled water, and dried to obtain alumina tungstic acid (ATA).

### Preparation of sulphated alumina tungstic acid (SATA)

Alumina tungstic acid (ATA) (2.5 g) was taken in a flat bottomed suction flask equipped with a dropping funnel, added CH_2_Cl_2_ (dry, 0.075 L) and stirred for 10 min. To the above reaction mixture, added dropwise chloro-sulfonic acid (1.75 mL) during 30 min. Upon complete addition, stirring of the reaction mixture was continued upto 90 min. HCl was removed under reduced pressure. Solid was filtered and thoroughly washed using CH_2_Cl_2_ as solvent and dried at 120 °C for 3 h to afford sulphated alumina tungstic acid (SATA).

### General procedure for synthesis of pyrazole carbonitrile derivatives

Equimolar quantity of aldehyde (1a–e) ethyl cyanoacetate (2), hydrazine (3a–b) and catalyst (SATA) (100 mg) in 5 mL ethanol was refluxed for an appropriate time (Table 2). After completion of the reaction (monitored by TLC) the reaction mixture was filtered off to remove the solid catalyst, which was washed with ethanol (3 × 15 mL) and ethyl acetate (2 × 10 mL) for further use. The filtrate was evaporated under reduced pressure to afford product (4). The crude product (4) was purified by recrystallization using ethanol/DMSO as solvent.

## Conflicts of interest

The authors declare no conflict of interest.

## Supplementary Material

RA-010-C9RA09013D-s001
