# Supplementary material for: Sulphated alumina tungstic acid (SATA): a highly efficient and novel heterogeneous mesostructured catalyst for the synthesis of pyrazole carbonitrile derivatives and evaluation of green metrics
Source: RSC Adv. 2020 Jan 3;10(2):818–27. doi: 10.1039/c9ra09013d (PMC9047446; doi:10.1039/c9ra09013d)
Supplement: RA-010-C9RA09013D-s001 [file RA-010-C9RA09013D-s001.pdf]

Electronic Supplementary Material (ESI) for RSC Advances.
This journal is © The Royal Society of Chemistry 2019

SUPPLEMENTARY INFORMATION

Sulphated alumina tungstic acid (SATA): A highly efficient and novel heterogeneous mesostructured catalyst for the synthesis of Pyrazole Carbonitrile Derivatives and evaluation of green metrics

Ryhan Abdullah Rather, Mohd Umar Khan and Zeba N. Siddiqui*

Department of Chemistry, Aligarh Muslim University, Aligarh, 202002, India

*Corresponding author. Email: *Siddiqui_zeba@yahoo.co.in*

Table of Contents

General Information 2

Spectroscopic data of compounds 3-6

Green metrics calculation 6-7

^{1}HNMR and ^{13}CNMR of Synthesized compounds 8-19

(I) General Information:

Melting points of all the synthesized compounds taken in a Riechert Thermover instrument and are uncorrected. The IR spectra were recorded on Perkin Elmer RXI spectrometer using KBr pellets, ^{1}H NMR, ^{13}C NMR spectra recorded on a Bruker DRX-400 spectrometer using tetramethylsilane (TMS) as an internal standard and DMSO-d6/$CDCl_3$ as solvent. Mass spectra were recorded on Micromass Quattro II (ESI) spectrometer. Elemental analyses (C, H and N) were conducted using the Elemental vario EL III elemental analyser and their results were found to be in agreement with the calculated values. TGA data was obtained with DSC-60 Shimadzu instrument and the analysis was performed in the temperature range of 0–600 ◦C at a constant heating rate of 20◦C/min in the nitrogen atmosphere. X-ray diffractograms (XRD) of the catalyst were recorded in the 2θ range of 20–80˚ with scan rate of 4◦/min on a Rigaku Minifax X-ray diffractometer with Ni-filtered Cu K_ radiation at a wavelength of 1.54060A˚. The SEM and EDX characterization of the catalyst were performed on QUANTA 200 FEG from FEI Netherlands. TEM analysis was performed on JEM-2100 F Model (ACC. Voltage: 200 kV) electron microscope. BET surface area of the sample was measured from the nitrogen adsorption/desorption isotherms obtained by using a Quantachrome Autosorb 1C BET analyzer at 77 K temperature. Prior to gas adsorption, the sample was degassed for 3 h at 423 K. All reagents were purchased from Merck, Aldrich and were used without further purification. The purity of compounds was checked by thin layer chromatography (TLC) on glass plates coated with silica gel G254 (E. Merck) using chloroform–methanol (3:1) mixture as mobile phase and visualized by iodine vapours and alcoholic ferric chloride.

(II) Spectral data of novel compounds:

1. **5-(4-chlorophenyl)-3-oxo-1-phenyl-2,3-dihydro-1H-pyrazole-4-carbonitrile:**
   White solid; Yield: 94%, m.p.170-172°C; Rf Value: 0.40 (10% methanol in chloroform); IR (KBr, cm^{-1}):, 3433, 2959, 2223, 1726, ^1H NMR (400 MHz, CDCl3): δ 3.97 (s, 1H, NH), 6.90 (s, 1H, ArH), 7.26 (s, 1H, ArH),), 7.47 (d, J = 4Hz, 2H, ArH), 7.92 (d, J = 4Hz, 2H, ArH), 8.22 (d, J = 8Hz, 2H, ArH) ppm. 13C NMR (400MHz, $CDCl_3$): 53.38, 103.18, 115.23, 120.35, 122.15, 130.15, 132.33, 139.63, 144.36, 151.28, 153.85, 162.97, 165.52, 174.25, 182.61 ppm. Anal. Found ($C_{16}H_{10}ClN_3O$) ; Calcd: C, 64.98; H, 3.41; N, 14.21.Found: C, 64.96; H, 3.43; N, 14.23 ESI-MS m/z ($C_{16}H_{10}ClN_3O + H^+$) ; Calcd: 296.0585; Found: 296.0671.

2. **5-(3-nitrophenyl)-3-oxo-1-phenyl-2,3-dihydro-1H-pyrazole-4-carbonitrile**
   Yellow solid;Yield: 94%, m.p.172-174°C; Rf Value: 0.38 (10% methanol in chloroform); IR (KBr, cm^{-1}):,3309, 2955, 2226, 1729, ^1H NMR (400 MHz, CDCl3): δ 4.38 (s, 1H, NH), 7.21(d, J = 4 Hz, 1H, ArH), 7.65-7.75 (m, 3H, ArH), 8.28-8.42 (m, 3H, ArH), 8.66 (s, 1H, ArH) ppm. 13C NMR (400MHz, $CDCl_3$): 66.34, 113.04, 119.86, 124.26, 125.42, 129.23, 130.13, 134.84, 137.60, 144.15, 154.12, 162.50, 164.99, 176.52, 179.80 ppm. Anal. Found ($C_{16}H_{10}N_4O_3$) ; Calcd: C, 62.74; H, 3.29; N, 18.29.Found: C, 62.76; H, 3.27; N, 18.31 ESI-MS m/z ($C_{16}H_{10}N_4O_3 + H^+$): 307.0826; Found: 307.0877.

3. **5-(4-nitrophenyl)-3-oxo-1-phenyl-2,3-dihydro-1H-pyrazole-4-carbonitrile**
   Yellow solid; Yield: 94%, m.p.172-174°C; Rf Value: 0.38 (10% methanol in chloroform); IR (KBr, cm^{-1}):, 3301, 2944, 2225, 1721, ^1H NMR (400 MHz, CDCl3): δ 4.45 (s, 1H, NH), 7.81 (d, J = 8Hz, 2H, ArH), 8.11-8.27 (m, 5H, ArH), 8.37 (d, J = 4Hz, 2H, ArH) ppm. 13C NMR (400MHz, $CDCl_3$): 63.34, 107.55, 112.68, 126.17, 130.89, 131.28, 137.07, 144.01, 149.87, 152.04, 160.79, 173.50, 182.23 ppm. Anal. Found ($C_{16}H_{10}N_4O_3$) ; Calcd: C, 62.74; H, 3.29; N, 18.29.Found: C, 62.76; H, 3.27; N, 18.31 ESI-MS m/z ($C_{16}H_{10}N_4O_3 + H^+$): 307.0826; Found: 307.1067.

4. **5-(3-chlorophenyl)-3-oxo-1-phenyl-2,3-dihydro-1H-pyrazole-4-carbonitrile**

White solid; Yield: 92%, m.p.170-172°C; Rf Value: 0.40 (10% methanol in chloroform); IR (KBr, cm^{-1}):, 3430, 2925, 2219, 1724, ^{1}H NMR (400 MHz, CDCl3): δ 3.90 (s, 1H, NH), 6.88 (d, J = 8Hz, 2H, ArH), 7.46-7.73 (m, 7H, ArH) ppm. 13C NMR (400MHz, $CDCl_3$): 55.26, 112.22, 113.98, 119.78, 121.79, 127.29, 129.31, 131.05, 131.89, 137.68, 144.31, 146.12, 146.65, 160.00, 168.06, 169.79, 180.49 ppm. Anal. Found ($C_{16}H_{10}ClN_3O$) ; Calcd: C, 64.98; H, 3.41; N, 14.21.Found: C, 64.97; H, 3.42; N, 14.23 ESI-MS m/z ($C_{16}H_{10}ClN_3O + H^+$) ; Calcd: 296.0585; Found: 296.0594.

5. **5-(4-bromophenyl)-3-oxo-1-phenyl-2,3-dihydro-1H-pyrazole-4-carbonitrile**

   Brown solid; Yield: 94%, m.p.164-166°C; Rf Value: 0.42 (10% methanol in chloroform); IR (KBr, cm^{-1}):, 3322, 2959, 2218, 1720, ^{1}H NMR (400 MHz, CDCl3): δ 4.40 (s, 1H, NH), 7.28 (s, 1H, ArH), 7.51 (d, J = 8Hz, 2H, ArH), 7.71 (d, J = 8Hz, 2H, ArH), 7.96 (d, J = 8Hz, 2H, ArH), 8.24 (d, J = 4Hz, 2H, ArH), ppm. 13C NMR (400MHz, $CDCl_3$): 55.55, 98.16, 115.24, 116.37, 123.60, 124.31, 125.48, 133.83, 142.19, 146.56, 154.29, 155.29, 162.96, 164.76, 171.68, 175.03, 180.42 ppm. Anal. Found ($C_{16}H_{10}BrN_3O$); Calcd: C, 56.48; H, 2.96; N, 12.35.Found: C, 56.46; H, 2.98; N, 12.37. ESI-MS m/z ($C_{16}H_{10}BrN_3O+H^+$) : Calcd: 340.0800; Found: 340.0854.

6. **5-(4-methoxyphenyl)-3-oxo-1-phenyl-2,3-dihydro-1H-pyrazole-4-carbonitrile**

   Violet solid; Yield: 92%, m.p.106-108°C; Rf Value: 0.40 (10% methanol in chloroform); IR (KBr, cm^{-1}):,3314, 2953, 2215, 1718, ^{1}H NMR (400 MHz, CDCl3): δ 3.88 (s, 3H, CH_3), δ 4.22 (s, 1H, NH), 6.94 (d, J = 4Hz, 2H, ArH), δ 7.30-7.84 (m, 9H, ArH) ppm. 13C NMR (400MHz, $CDCl_3$): 52.99, 58.80, 98.85, 108.69, 114.19, 116.37, 123.95, 126.52, 133.83, 138.20, 151.28, 153.85, 163.33, 165.91, 170.27, 179.68, 188.10 ppm. Anal. Found ($C_{17}H_{13}N_3O_2$); Calcd: C, 70.08; H, 4.50; N, 14.41; Found: C, 70.10; H, 4.52; N, 14.39. ESI-MS m/z ($C_{17}H_{13}N_3O_2+H^+$):Calcd: 292.1081; Found: 292.1074.

7. **5-(4-chlorophenyl)-3-oxo-2,3-dihydro-1H-pyrazole-4-carbonitrile**

   White solid;Yield: 94%, m.p.170-172°C; Rf Value: 0.38 (10% methanol in chloroform); IR (KBr, cm^{-1}):, 3433, 2958, 2222, 1726, ^{1}H NMR (400 MHz, CDCl3): δ 3.96 (d, J =4 Hz, 1H, NH^a), δ 4.36 (d, J =4 Hz, 1H, NH^b), 7.43(d, J = 4Hz, 2H, ArH), 7.95(d, J = 8Hz, 2H, ArH) ppm. 13C NMR (400MHz, $CDCl_3$): 53.66, 63.14, 103.19, 114.85, 129.47, 132.27, 140.01, 154.25, 152.22, 171.70 ppm. Anal. Found ($C_{10}H_6ClN_3O$);Calcd: C,

54.69; H, 2.75; N, 19.13; Found: C, 54.71; H, 2.73; N, 19.15. ESI-MS m/z ($C_{10}H_6ClN_3O$ + H $^+$); Calcd: 220.0272; Found: 220.0252.

8. 5-(3-nitrophenyl)-3-oxo-2,3-dihydro-1H-pyrazole-4-carbonitrile

yellow solid;Yield:92%, m.p.172-174°C; Rf Value: 0.38 (10% methanol in chloroform); IR (KBr, cm^{-1}):, 3428, 2965, 2220, 1724, ^{1}H NMR (400 MHz, CDCl3): δ 4.03 (d, 1H, J = 4 Hz, NH^a), δ 4.46 (d, J = 4Hz, 1H, NH^b), 7.31 (s, 1H, ArH), 7.71 (s, 1H, ArH), 8.37 (d, J= 8 Hz, 1H, ArH), 8.75 (s, 1H, ArH) ppm. 13C NMR (400MHz, $CDCl_3$):): 63.20, 106.82, 114.78, 126.22, 127.29, 130.58, 132.65, 134.94, 148.97, 151.77, 161.68 ppm. Anal. Found ($C_{10}H_6N_4O_3$); Calcd: C, 52.18; H, 2.63; N, 24.34; Found: C, 52.16; H, 2.61; N, 24.35. ESI-MS m/z ($C_{10}H_6N_4O_3$+H$^+$); Calcd: 231.0513;Found:231.0535.

9. 5-(4-nitrophenyl)-3-oxo-2,3-dihydro-1H-pyrazole-4-carbonitrile

yellow solid; Yield: 94%, m.p.172-174°C; Rf Value: 0.38 (10% methanol in chloroform); IR (KBr, cm^{-1}):, 2928, 2208, 1640,1580, 1510, 1340, 1248, ^{1}H NMR (400 MHz, CDCl3): δ 3.95 (d, J = 4 Hz, 1H, NH^a), δ 4.40 (d, J = 4 Hz, 1H, NH^b), 7.23 (s, 1H, ArH), 8.10-8.36 (m, 4H, ArH) ppm. 13C NMR (400MHz, $CDCl_3$):63.20, 115.00, 126.03, 127.49, 130.38, 133.11, 134.42, 147.90, 151.97, 161.68 ppm. Anal. Found ($C_{10}H_6N_4O_3$);Calcd: C, 52.18; H, 2.63; N, 24.34. Found: C, 52.17; H, 2.61; N, 24.35. ESI-MS m/z ($C_{10}H_6N_4O_3$+ H$^+$): 231.0513;Found: 231.0563.

10. 5-(3-chlorophenyl)-3-oxo-2,3-dihydro-1H-pyrazole-4-carbonitrile

White solid; Yield: 92%, m.p.172-174°C; Rf Value: 0.38 (10% methanol in chloroform); IR (KBr, cm^{-1}):,3424, 2945, 2225, 1722, ^{1}H NMR (400 MHz, CDCl3): δ 4.02 (d, J = 4 Hz, 1H, NH^a), δ 4.46 (d, J = 4 Hz, 1H, NH^b), 8.34-8.57 (m, 3H, ArH), 8.75 (s, 1H, ArH) ppm. 13C NMR (400MHz, $CDCl_3$): 64.00, 114.54, 126.03, 126.49, 130.58, 132.09, 135.26, 148.44, 153.02, 160.93 ppm. Anal. Found ($C_{10}H_6ClN_3O$); Calcd: C, 54.69; H, 2.75; N, 19.13; Found: C, 54.71; H, 2.73; N, 19.15. ESI-MS m/z ($C_{10}H_6ClN_3O$ + H $^+$); Calcd: 220.0272; Found: 220.0262.

11. 5-(4-bromophenyl)-3-oxo-2,3-dihydro-1H-pyrazole-4-carbonitrile

Brown solid;Yield: 92%, m.p.162-164°C; Rf Value: 0.38 (10% methanol in chloroform); IR (KBr, cm^{-1}):, 3432, 2928, 2220, 1720, ^{1}H NMR (400 MHz, CDCl3): δ 3.96 (d, J = 4 Hz, 1H, NH^a), δ 4.46 (d, J = 4Hz, 1H, NH^b), 7.57 (d, J = 4 Hz, 2H, ArH), 7.87 (d, J = 4 Hz, 2H, ArH), 8.20 (s, 1H, ArH) ppm. 13C NMR (400MHz, $CDCl_3$): 63.47, 114.78,

125.75, 127.29, 130.11, 133.11, 135. 26,148.44, 151.97, 161.68 ppm. Anal. Found ($C_{10}H_6BrN_3O$);Calcd: C, 45.48; H, 2.29; N, 15.91; Found: C, 45.50; H, 2.27; N, 15.89.ESI-MS m/z ($C_{10}H_6BrN_3O + H^+$); Calcd: 263.9767; Found: 263.9786.

12. 5-(4-methoxyphenyl)-3-oxo-2,3-dihydro-1H-pyrazole-4-carbonitrile

Violet solid; Yield: 94%, m.p.106-108°C; Rf Value: 0.40 (10% methanol in chloroform); IR (KBr, cm^{-1}):,3435, 2999, 2221, 1725, ^{1}H NMR (400 MHz, CDCl3): δ 3.97 (s 3H, OCH3), δ 4.38 (d, J = 8 Hz, 1H, NH^a), δ 4.72 (d, J = 4 Hz, 1H, NH^b), 7.71 (s, 1H, ArH), 8.31 (d, J = 8Hz , 2H, ArH), 8.68 (s, 1H, ArH) ppm. 13C NMR (400MHz, $CDCl_3$): 52.99, 55.85, 99.58, 113.82, 117.05, 123.95, 127.28, 133.08, 154.22, 163.33, 185.55 ppm. Anal. Found ($C_{11}H_9N_3O_2$) ;Calcd: C, 61.39; H, 4.22; N, 19.41;Found: C, 61.41; H, 4.21; N, 19.43. ESI-MS m/z ($C_{11}H_9N_3O_2$+ H^+) : Calcd: 216.0768; Found: 216.0773.

III. Materials used for green metrics calculations

A. Formula used for calculations:

The following formulae were used for evaluating green chemistry metrics such as atom economy (AE), carbon efficiency (CE), reaction mass efficiency (RME), overall efficiency (OE), process mass intensity (PMI) solvent intensity (SI) and E-factor.[1,2]

***1. % Atom Economy (AE)** = (Mol Wt. of desired product/ Mol Wt. of all reactants) × 100*

***2. % Carbon Efficiency (CE)** = (Amount of carbon in the product)/ Total no of carbon present in reactant) × 100*

***3. % Reaction Mass Efficiency (RME)** = (Mass of isolated product)/ Total mass of reactants) × 100*

***4. % Overall Efficiency (OE)** = (RME/AE) × 100*

***5. % Process Mass Intensity (PMI)** = (Total mass of input materials in a process)/ Mass of product) × 100*

*6. **Solvent Intensity (SI)** = (Total mass of input solvents in a process)/ Mass of product)*

*7. **E-Factor** = PMI-1*

B. Details of reactants and products used for metric calculations:

a) Reactants: Ethylcyanoacetate, Molecular Weight = 113.11g/mol, weight used = 0.113 g; Phenylhydrazine, Molecular weight = 108.14 g/mol, weight used = 0.108g; Hydrazinehydrate, Molecular weight = 32.04 g/mol;

Weight of different aromatic aldehydes: 4-Cholorobenzaldehyde = 0.140g, 3-Cholorobenzaldehyde = 0.140g, 4-Nitrobenzaldehyde = 0.151g, 3-Nitrobenzaldehyde = 0.151g, 4-Bromobenzaldehyde = 0.185g, 4-Methoxybenzaldehyde = 0.136g.

b). Products:

**Compounds**	**4a**	**4b**	**4c**	**4d**	**4e**	**4f**	**4g**
**Molecular Formula**	$C_{16}H_{10}ClN_3O$	$C_{16}H_{10}N_4O_3$	$C_{16}H_{10}N_4O_3$	$C_{16}H_{10}ClN_3O$	$C_{16}H_{10}BrN_3O$	$C_{17}H_{13}N_3O_2$	$C_{10}H_6ClN_3O$
**Molecular Weight (g/mol)**	295.72	306.28	306.28	295.72	340.18	290.31	219.62
**Weight (g)**	0.278	0.288	0.288	0.273	0.320	0.268	0.207
**Yield (%)**	94	94	94	92	94	92	94

**Compounds**	**4h**	**4i**	**4j**	**4k**	**4l**
**Molecular Formula**	$C_{10}H_6N_4O_3$	$C_{10}H_6N_4O_3$	$C_{10}H_6ClN_3O$	$C_{10}H_6BrN_3O$	$C_{11}H_9N_3O_2$
**Molecular Weight (g/mol)**	230.18	230.18	219.62	264.08	215.21
**Weight (g)**	0.212	0.217	0.203	0.243	0.203
**%Yield**	92	94	92	92	94

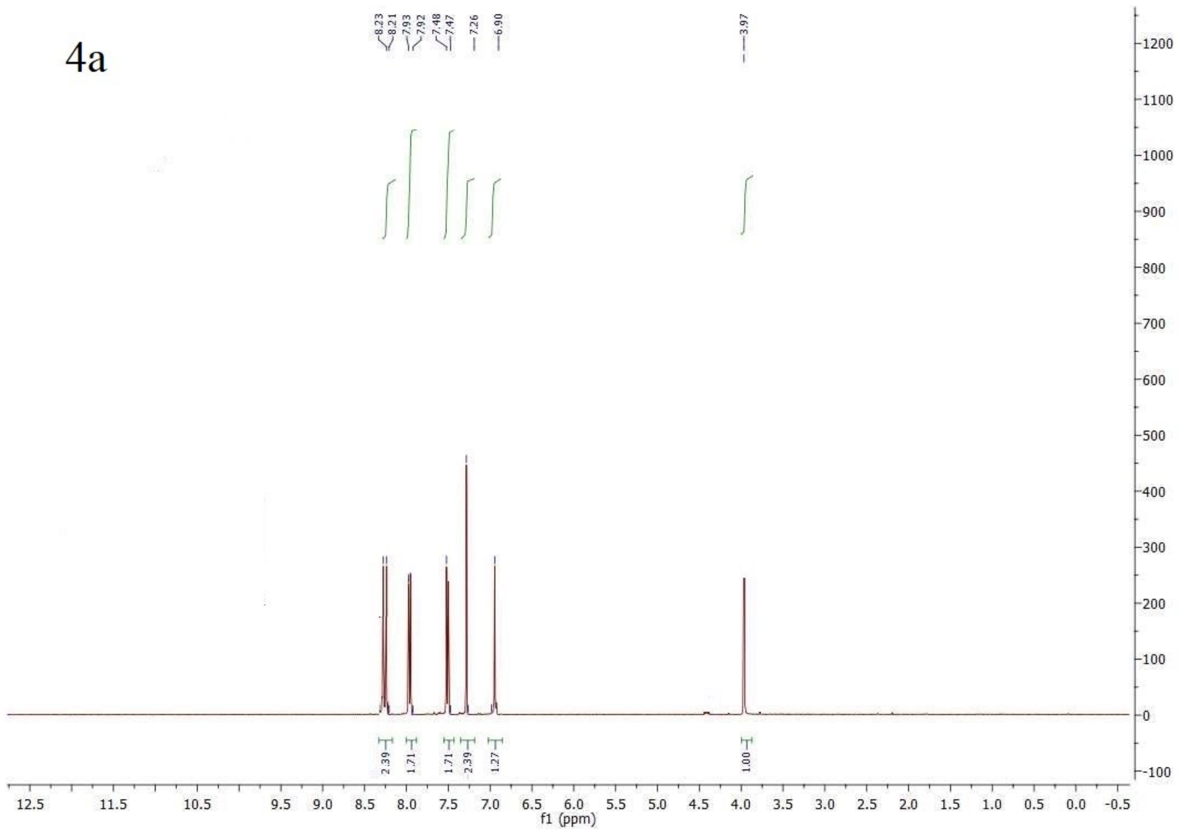

Figure S1.^{1}H NMR Spectrum of compound 4a

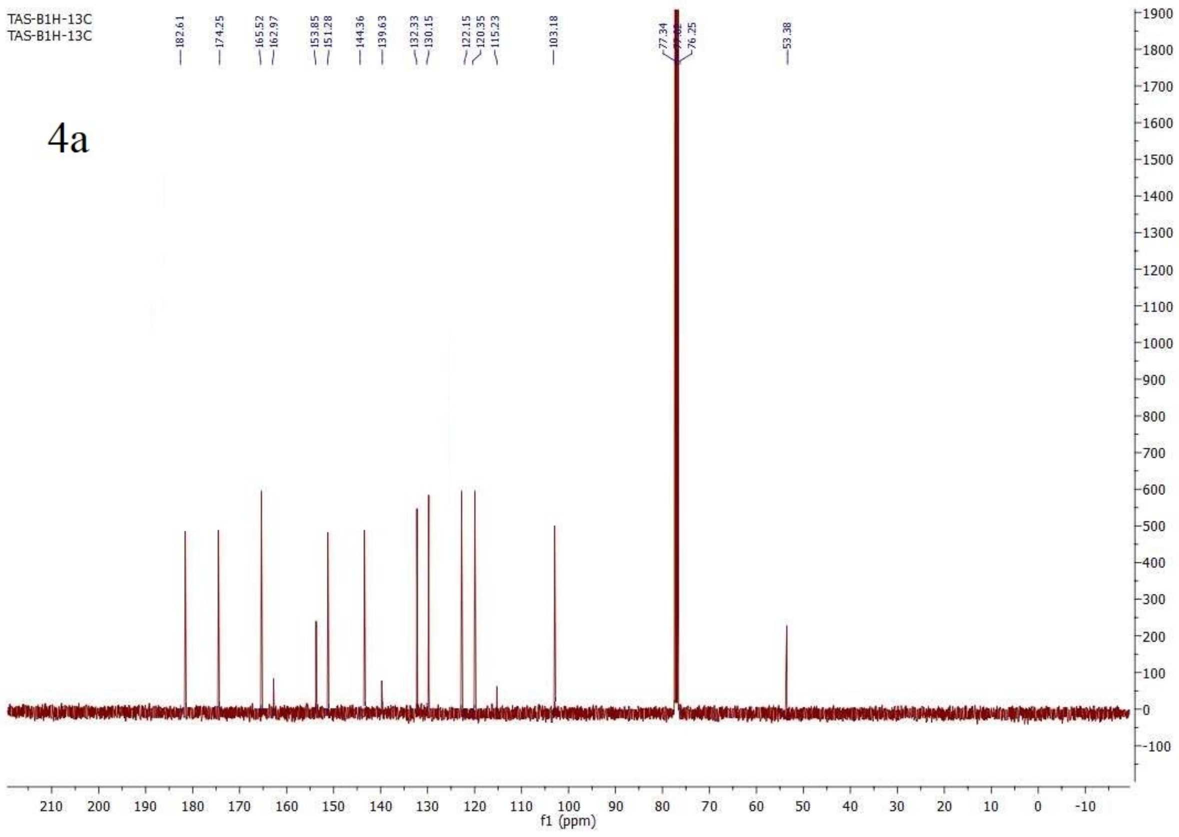

Figure S2.^{13}CNMR Spectrum of compound 4a

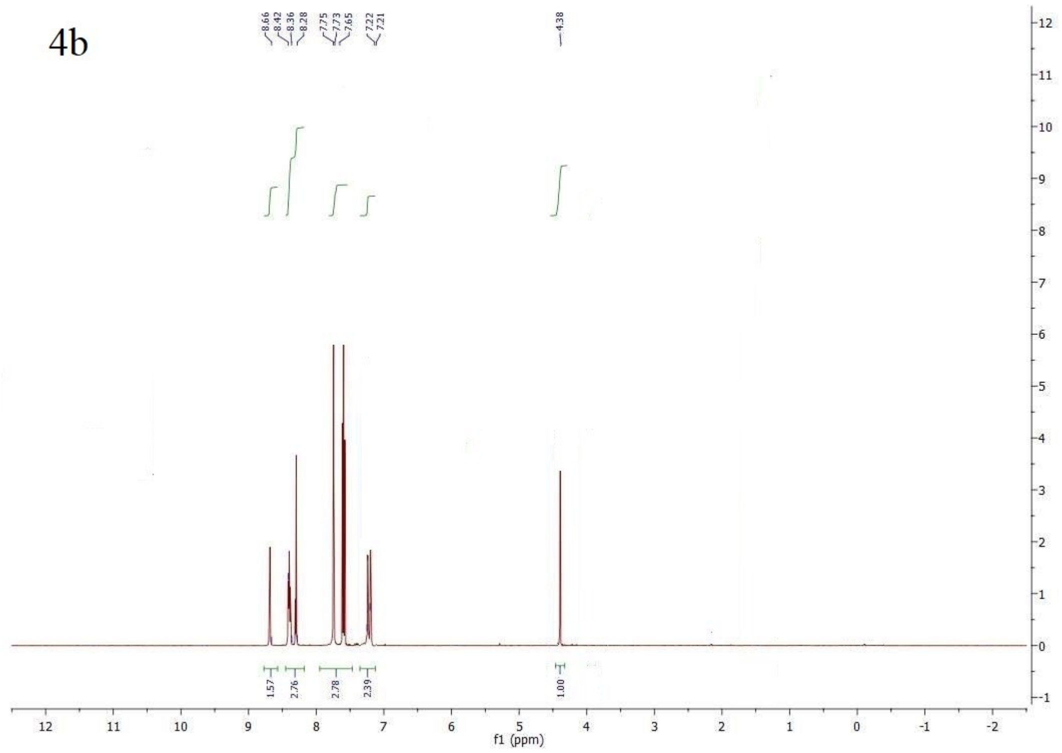

Figure S3.^{1}H NMR Spectrum of compound 4b

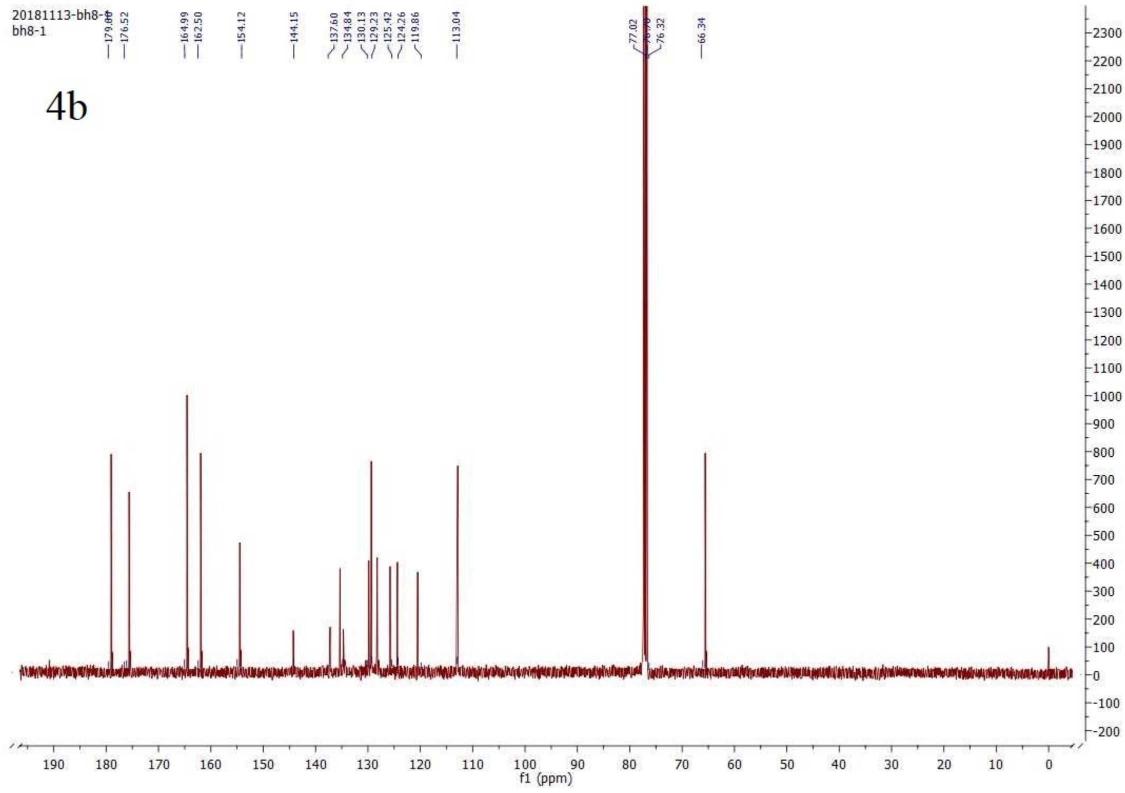

Figure S4.^{13}CNMR Spectrum of compound 4b

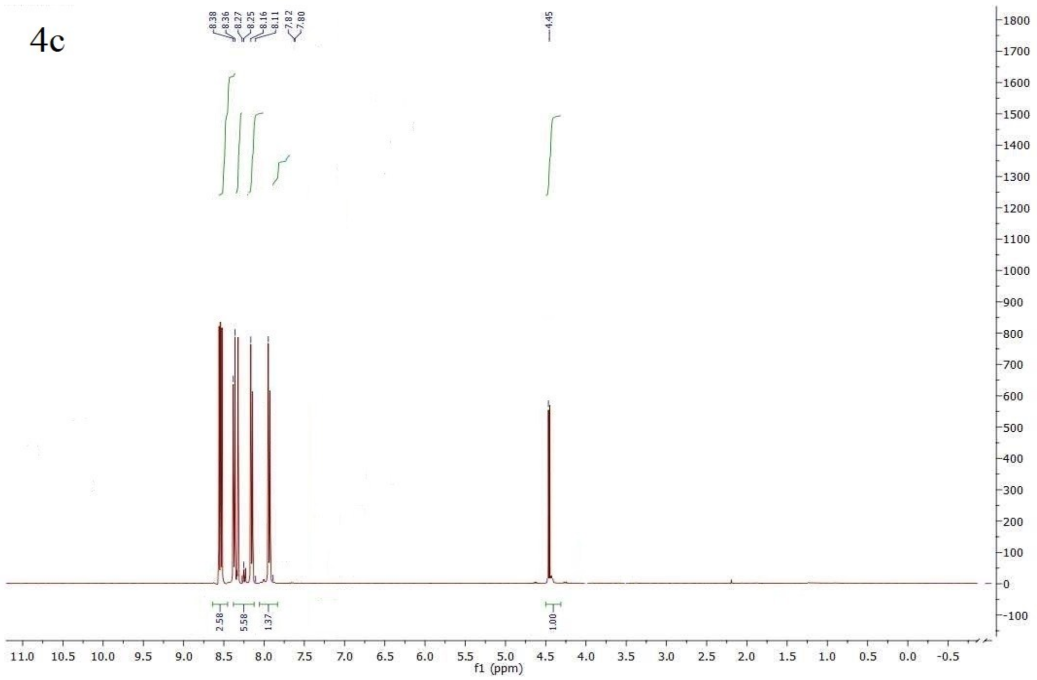

Figure S5.^{1}H NMR Spectrum of compound 4c

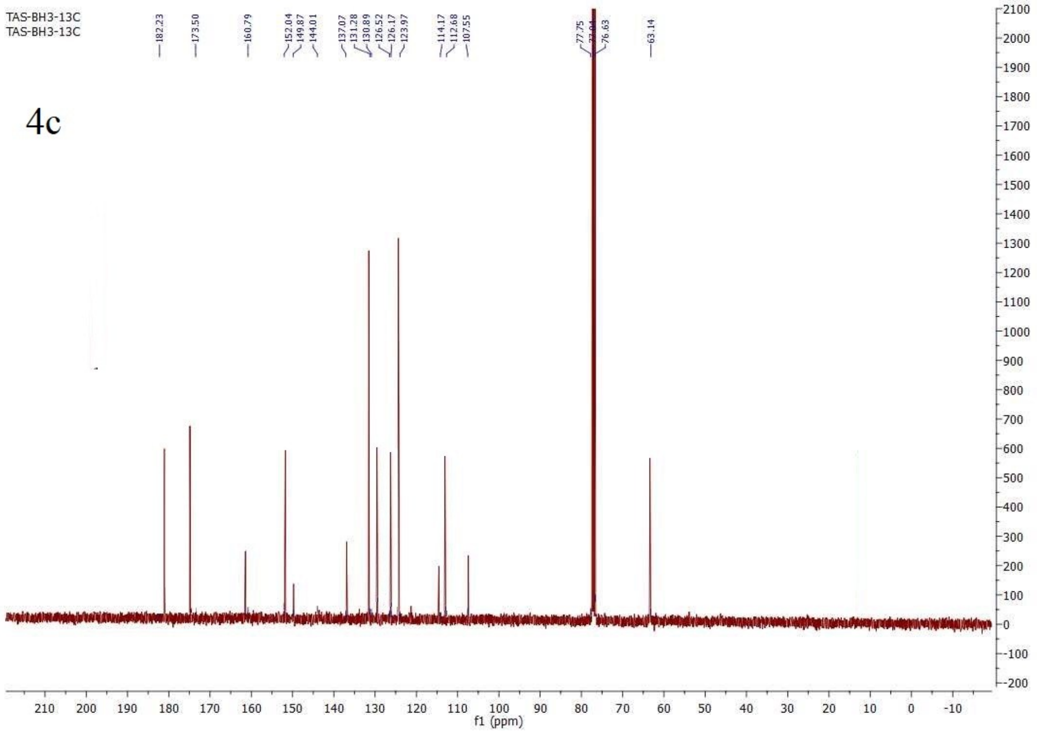

Figure S6.^{13}CNMR Spectrum of compound 4c

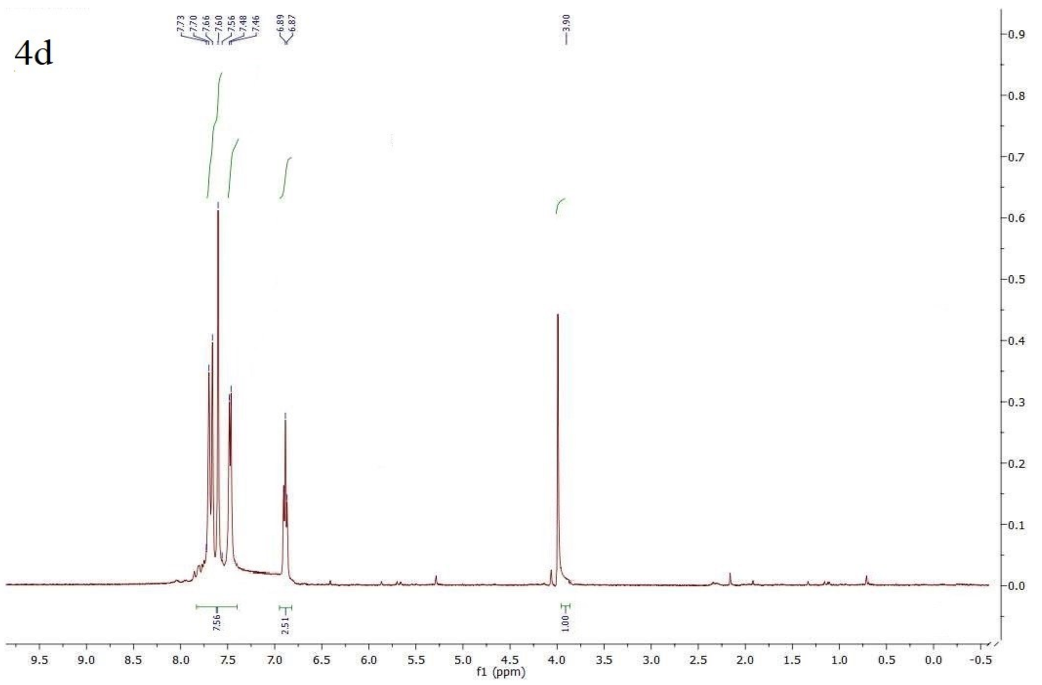

Figure S7.^{1}H NMR Spectrum of compound 4d

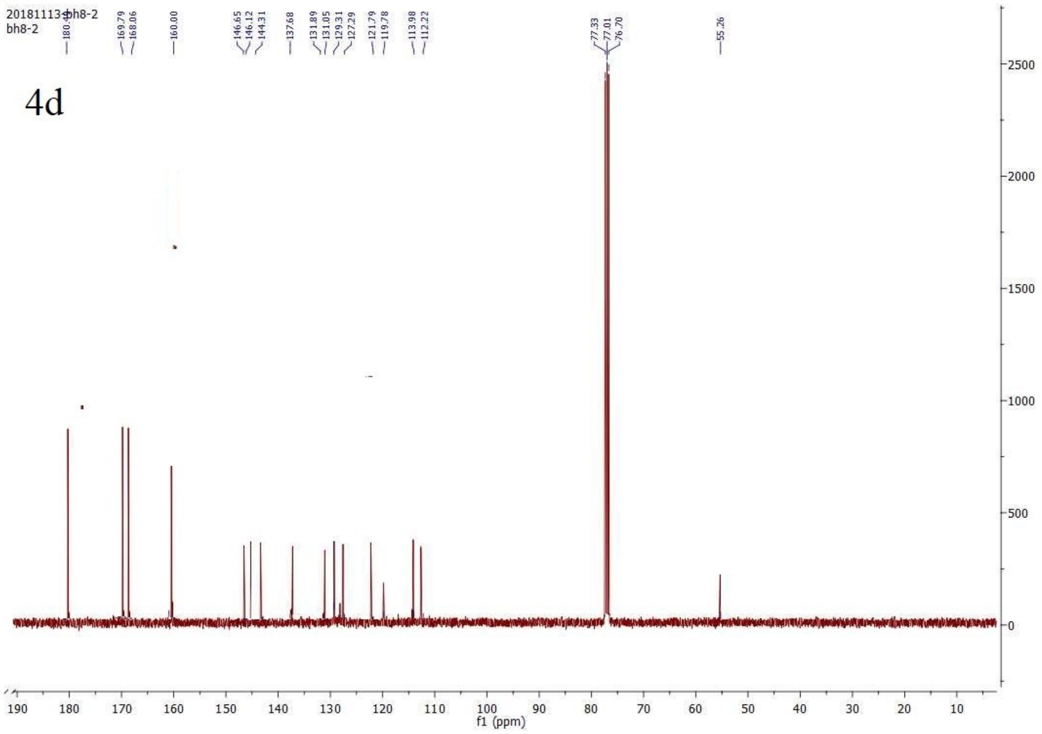

Figure S8.^{13}CNMR Spectrum of compound 4d

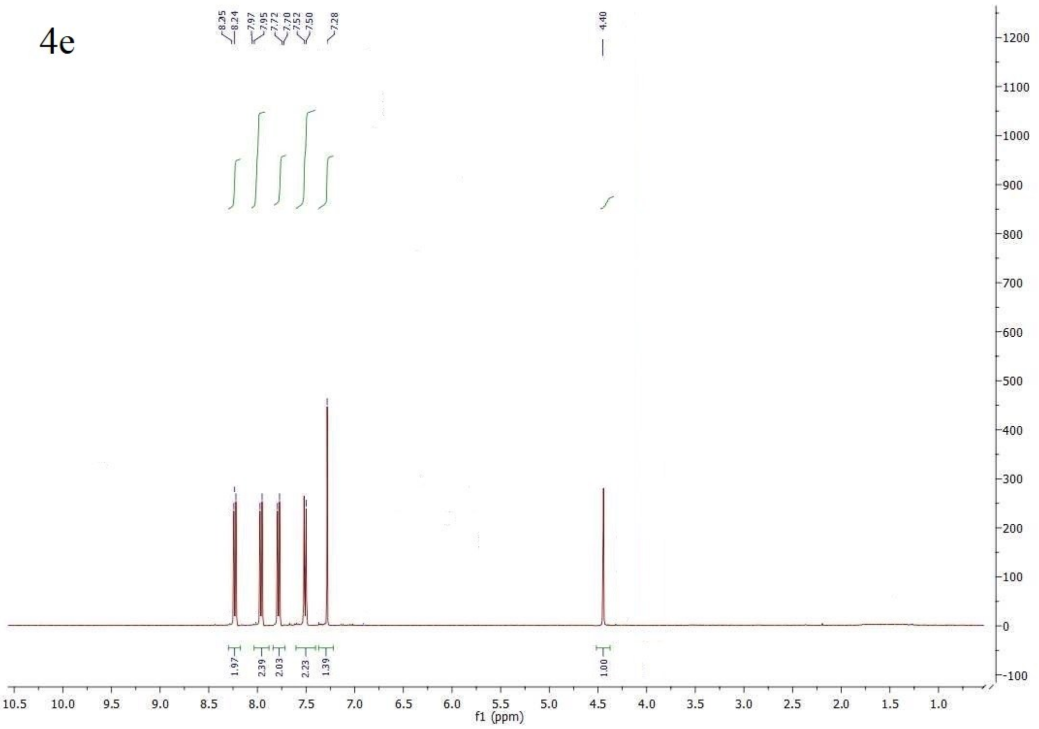

Figure S9.^{1}H NMR Spectrum of compound 4e

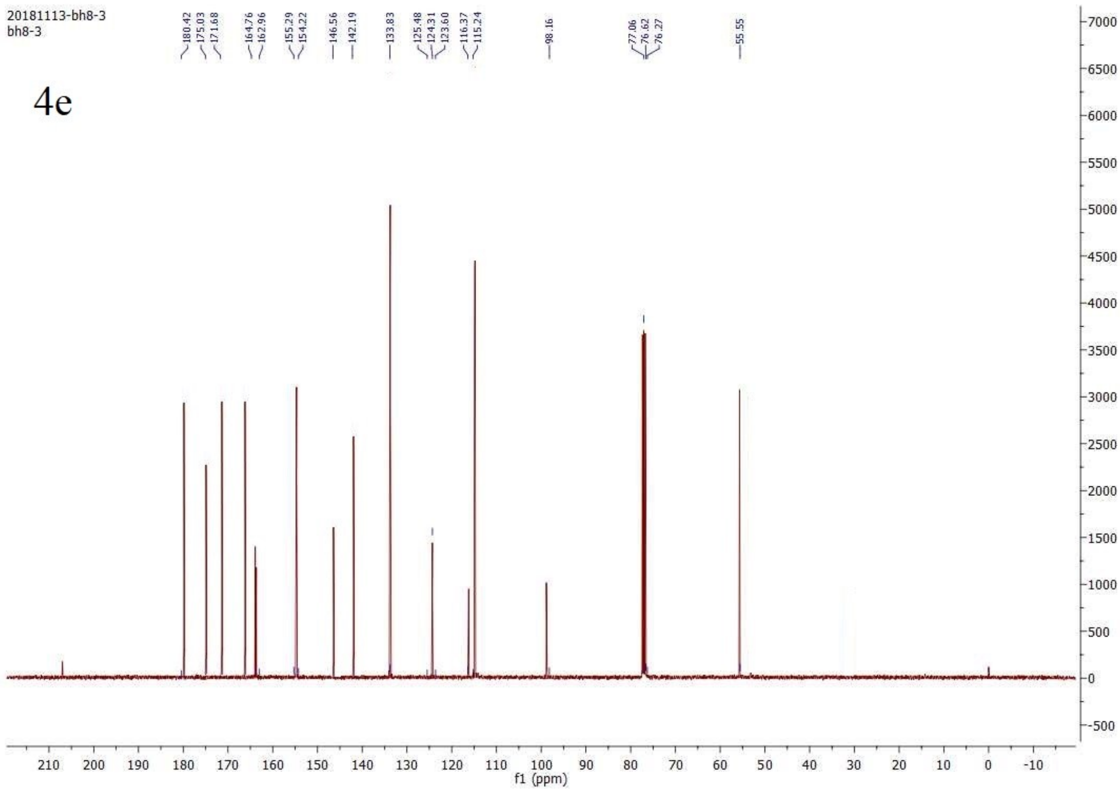

Figure S10.^{13}CNMR Spectrum of compound 4e

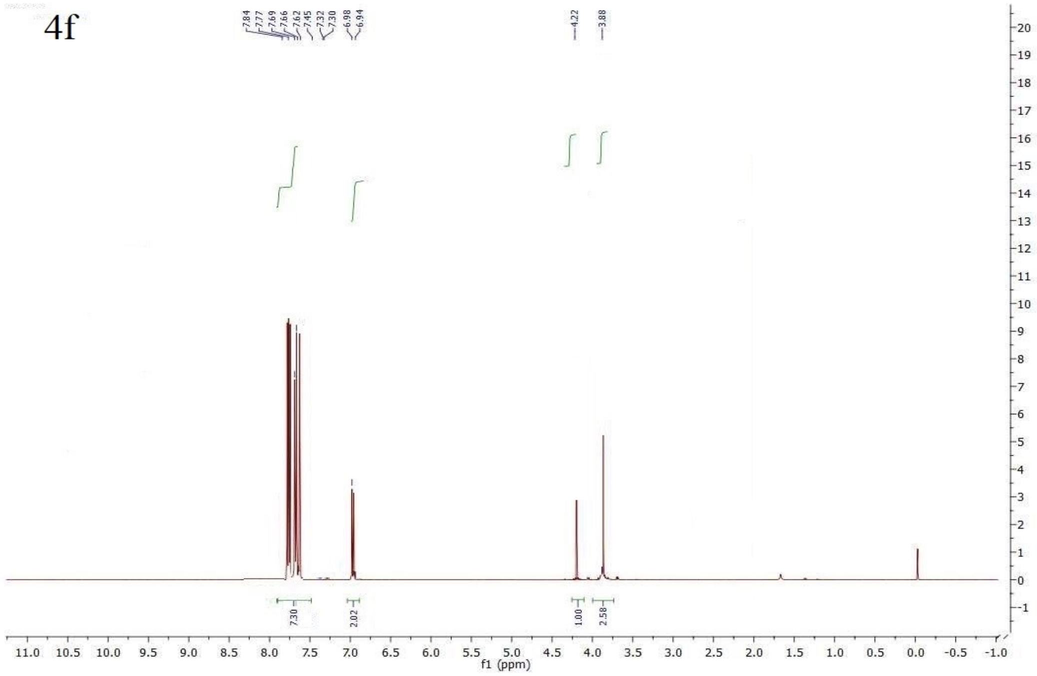

Figure S11.^{1}H NMR Spectrum of compound 4f

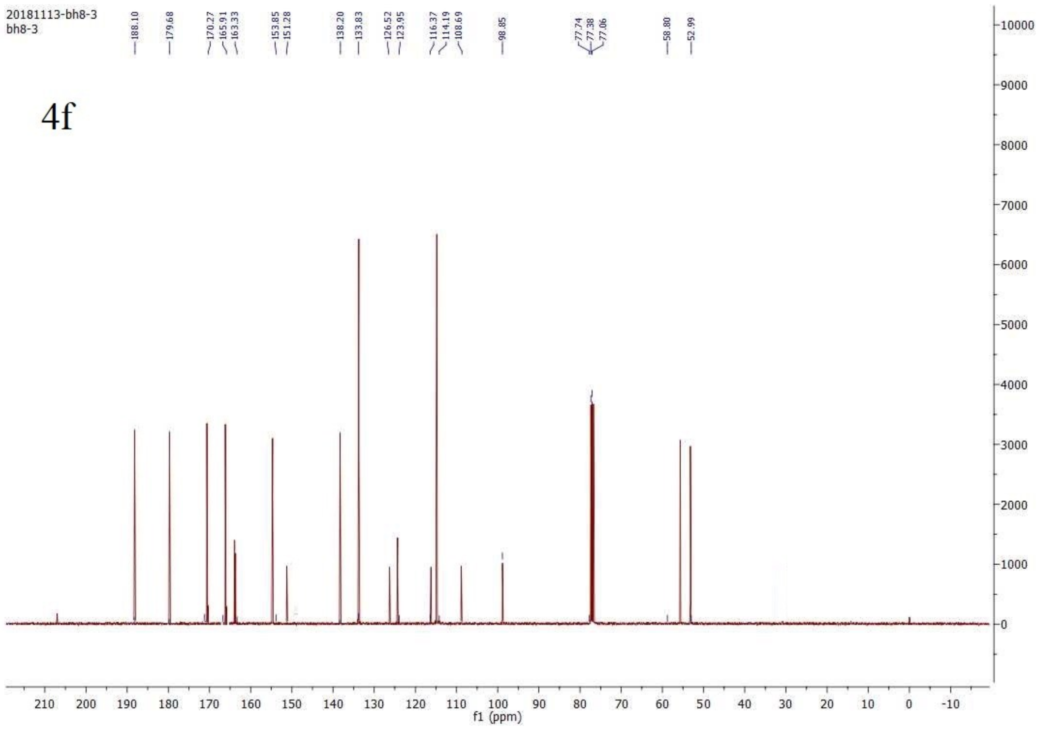

Figure S12.^{13}CNMR Spectrum of compound 4f

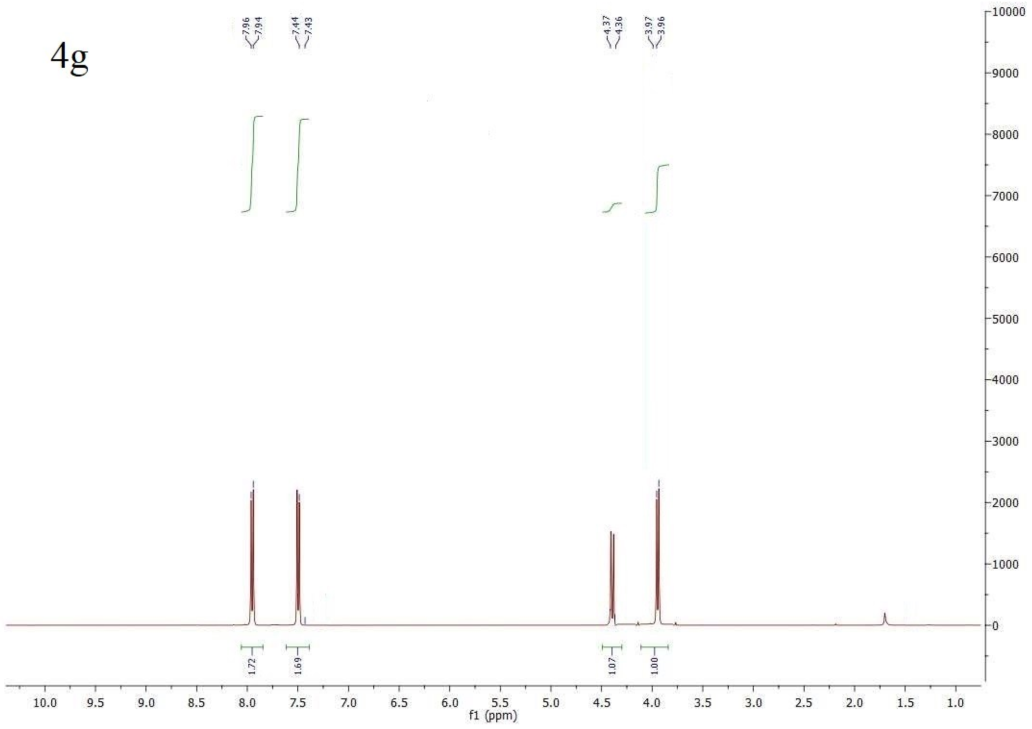

Figure S13.^{1}H NMR Spectrum of compound 4g

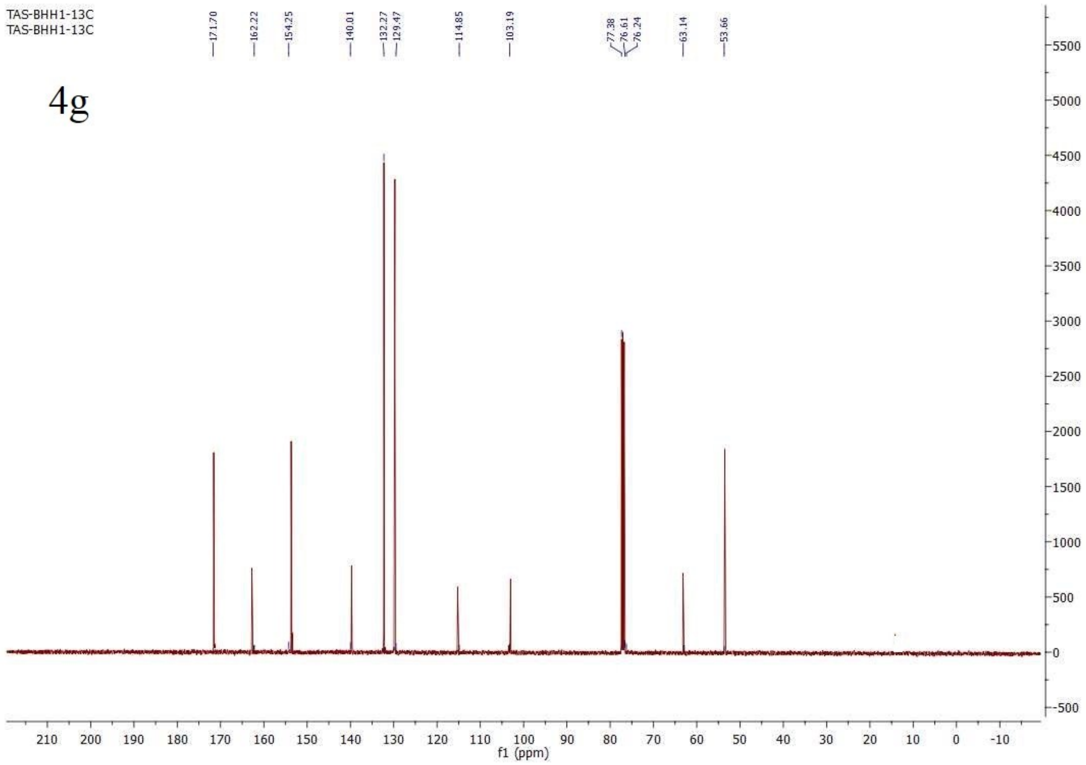

Figure S14.^{13}CNMR Spectrum of compound 4g

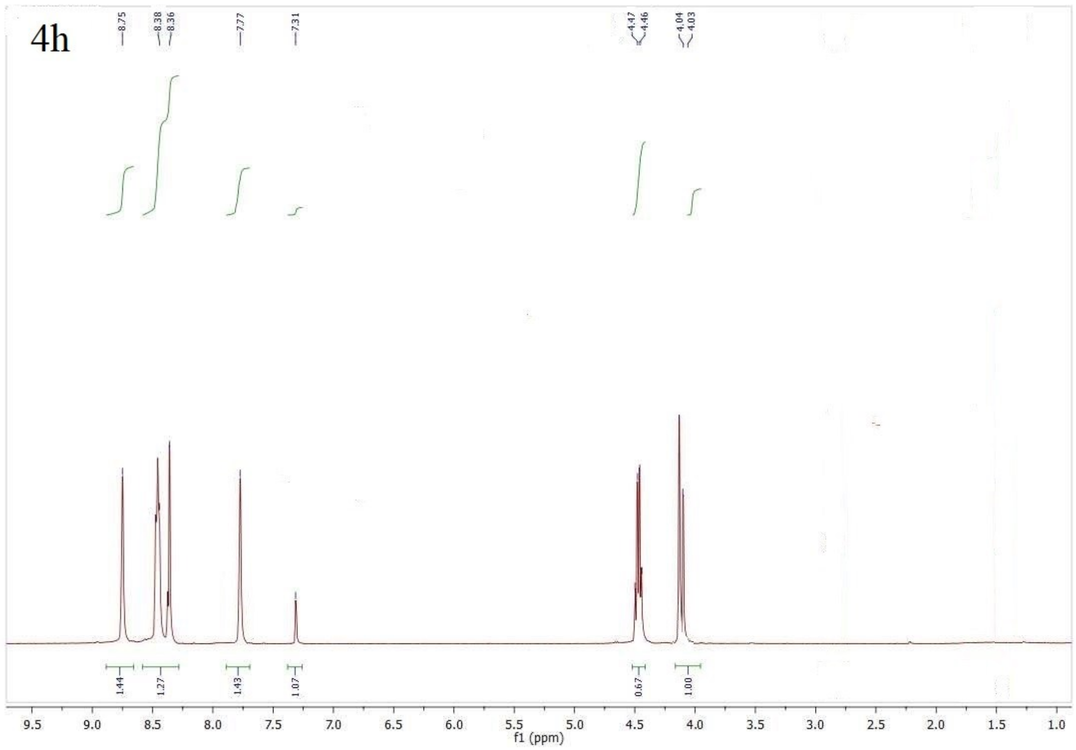

Figure S15.^{1}H NMR Spectrum of compound 4h

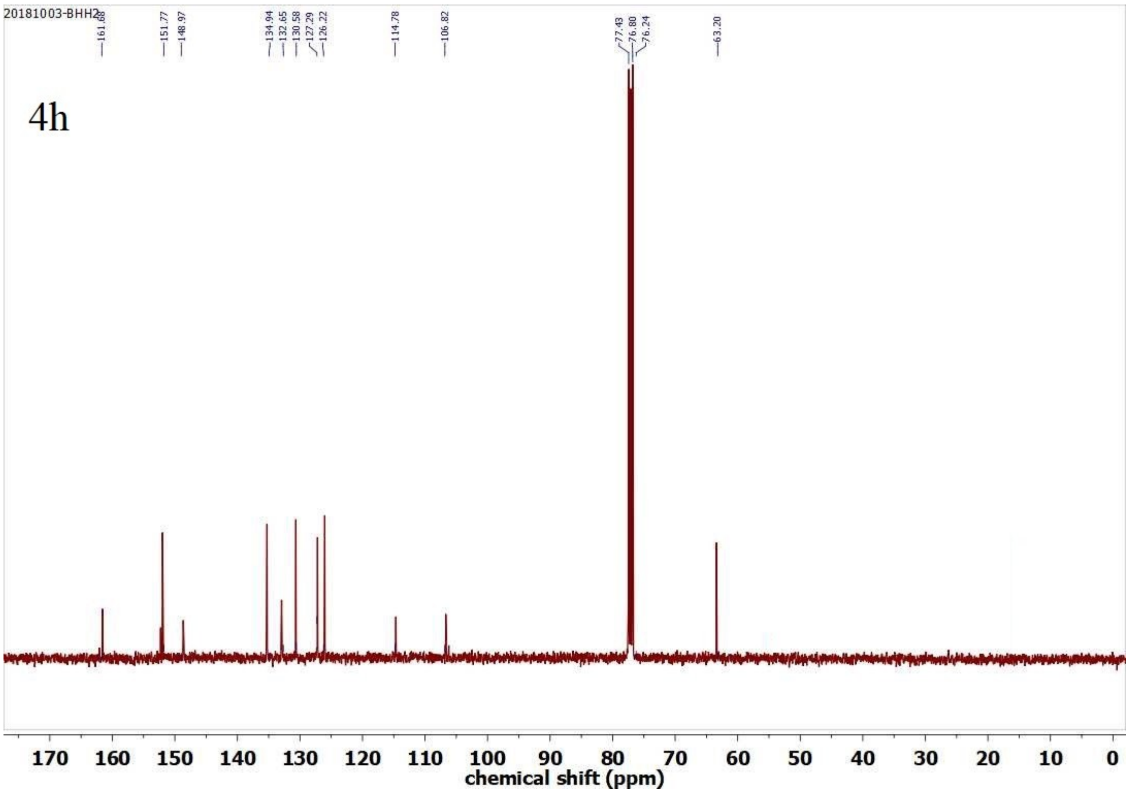

Figure S16.^{13}CNMR Spectrum of compound 4h

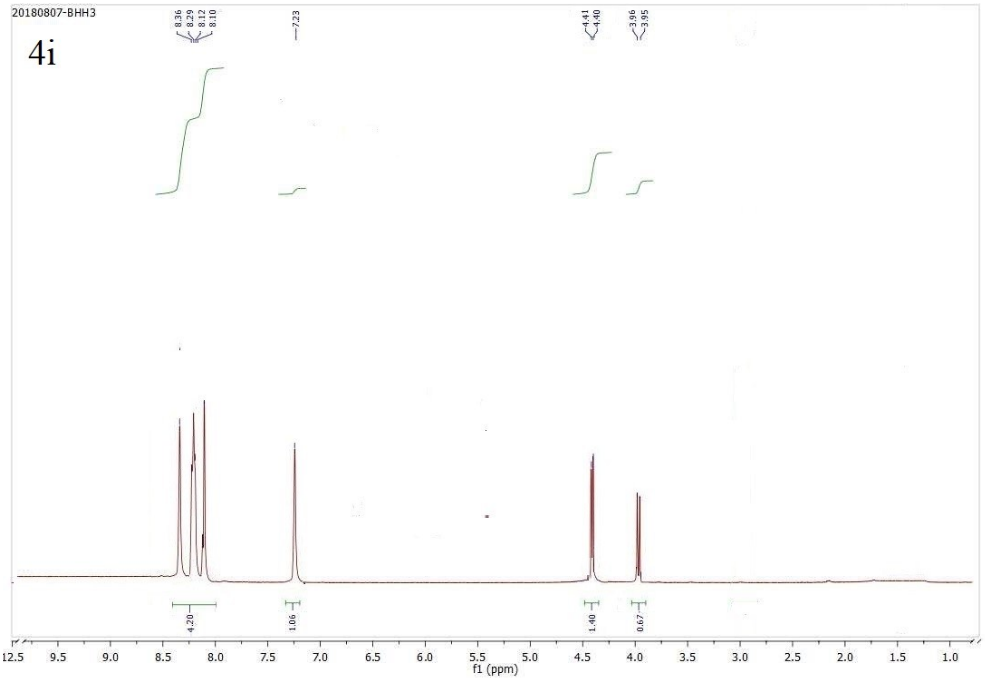

Figure S17.^{1}H NMR Spectrum of compound 4i

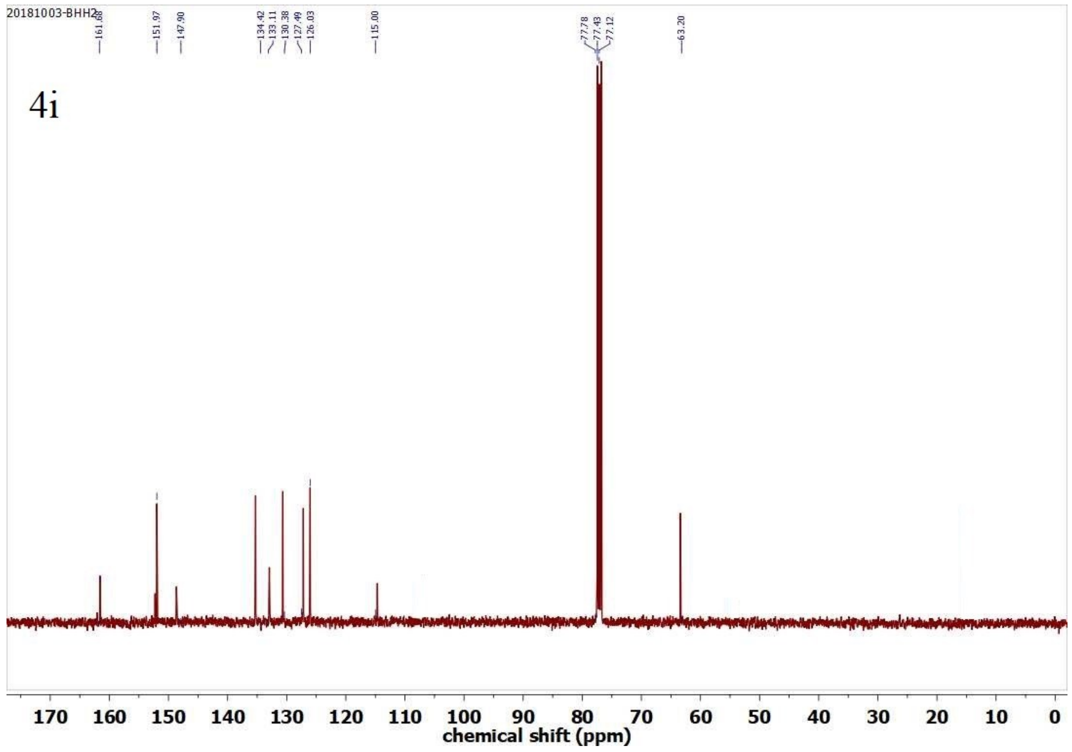

Figure S18.^{13}CNMR Spectrum of compound 4i

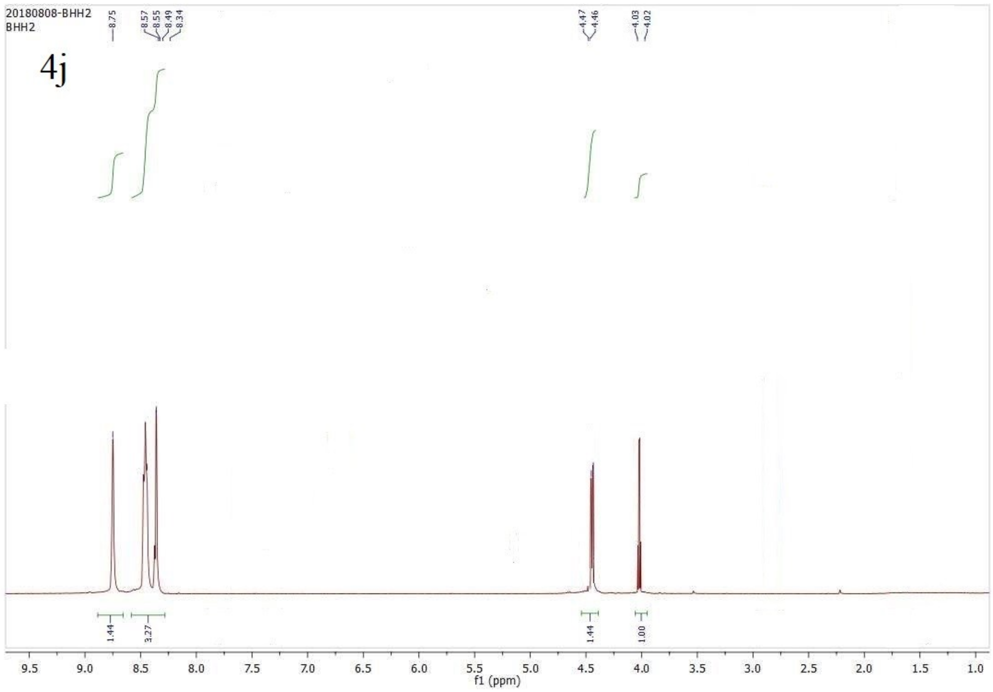

Figure S19.^{1}H NMR Spectrum of compound 4j

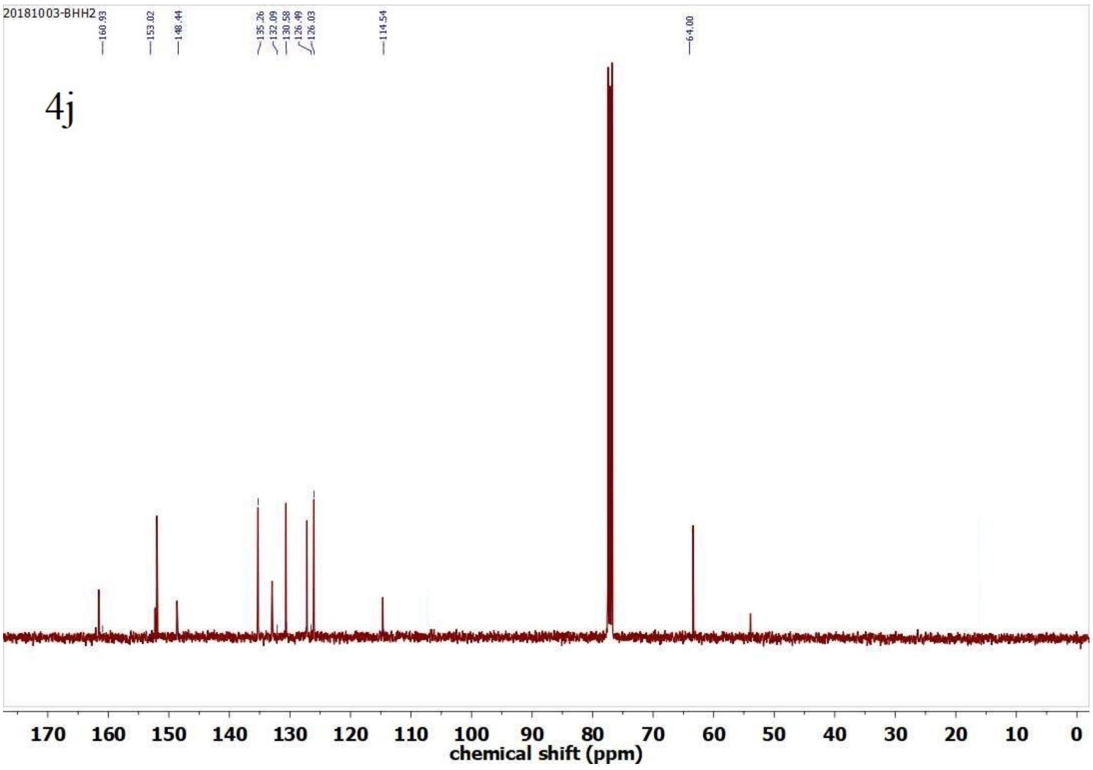

Figure S20.^{13}CNMR Spectrum of compound 4j

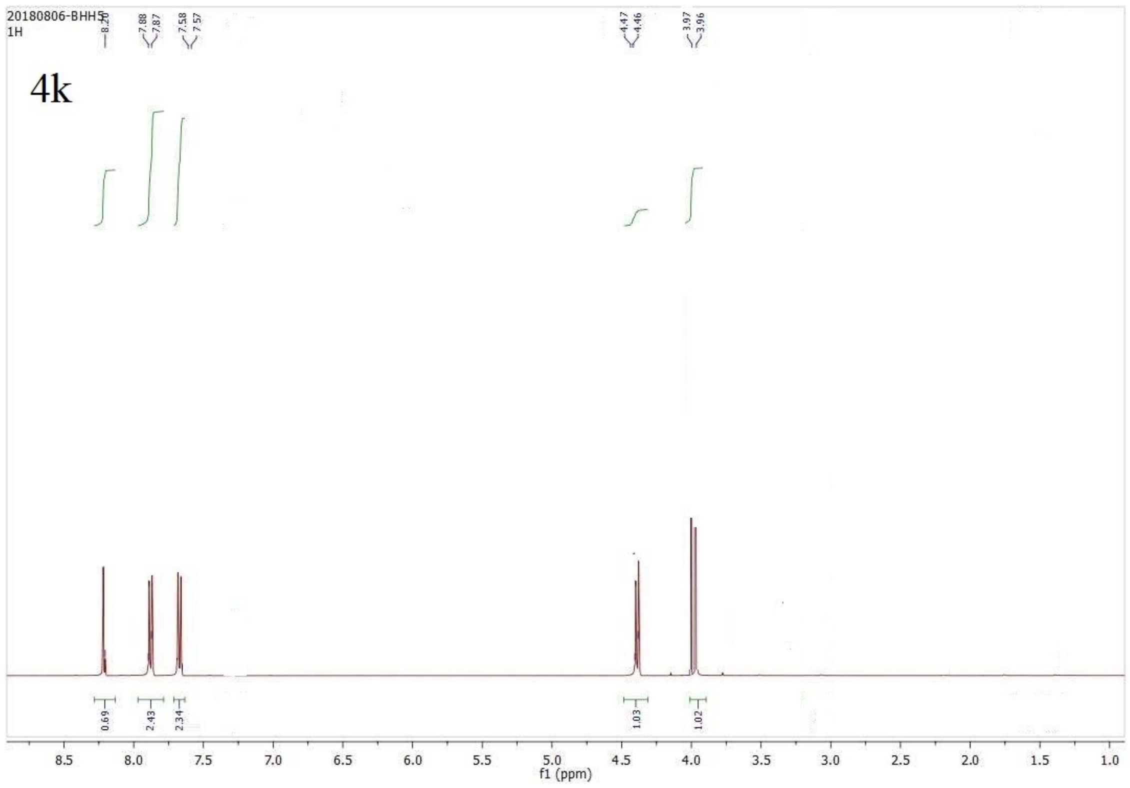

Figure S21.^{1}H NMR Spectrum of compound 4k

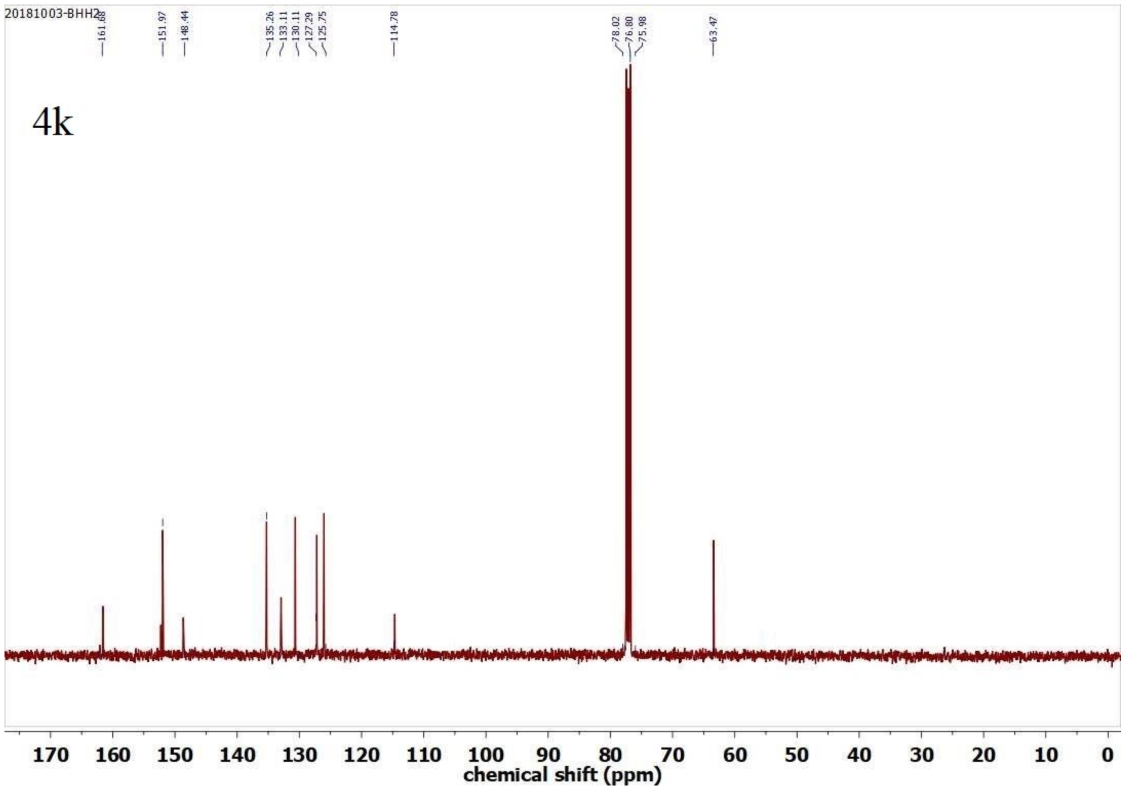

Figure S22.^{13}CNMR Spectrum of compound 4k

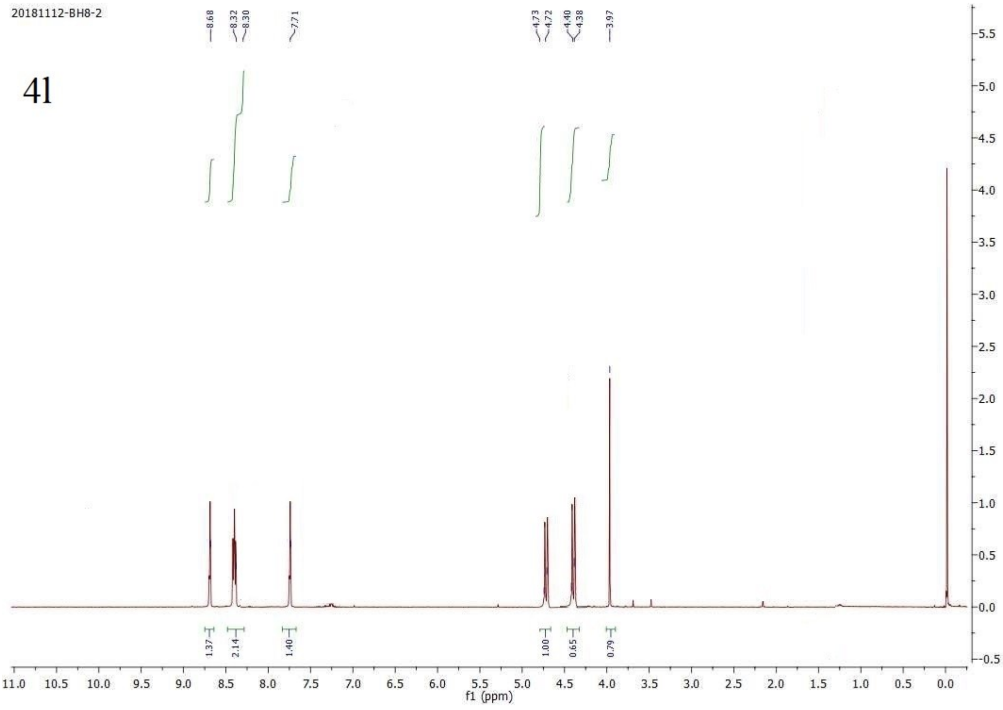

Figure S23.^{1}H NMR Spectrum of compound 4l

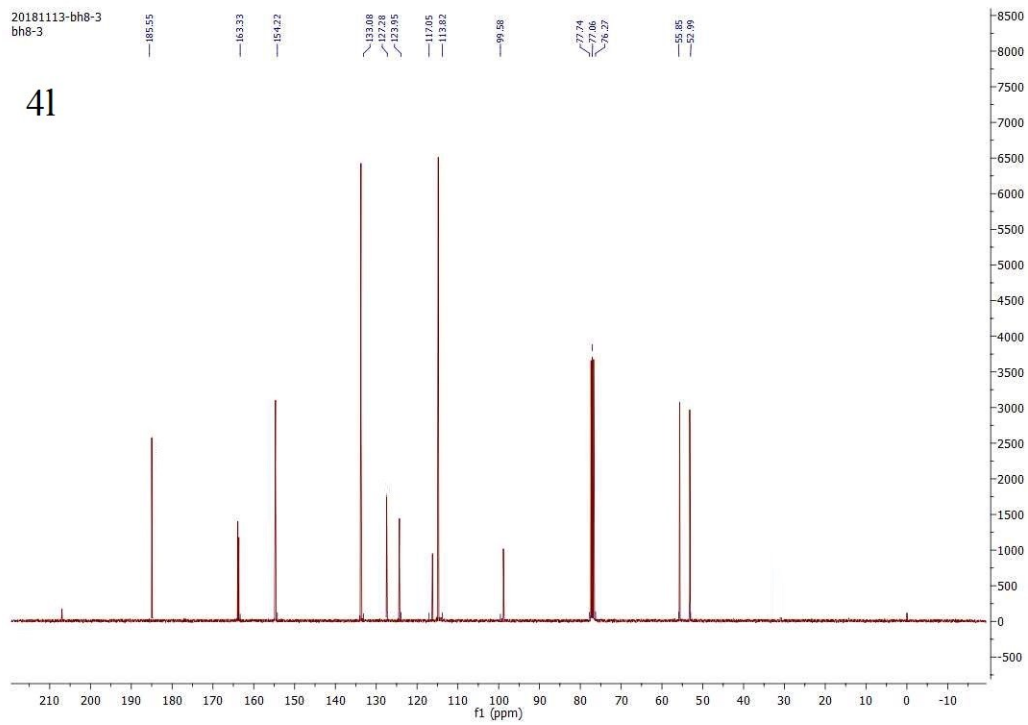

Figure S24.^{13}CNMR Spectrum of compound 4l
